# CFLAP1 and CFLAP2 Are Two bHLH Transcription Factors Participating in Synergistic Regulation of AtCFL1-Mediated Cuticle Development in *Arabidopsis*

**DOI:** 10.1371/journal.pgen.1005744

**Published:** 2016-01-08

**Authors:** Shibai Li, Xiaochen Wang, Shan He, Jieru Li, Qingpei Huang, Takato Imaizumi, Leqing Qu, Genji Qin, Li-Jia Qu, Hongya Gu

**Affiliations:** 1 State Key Laboratory for Protein and Plant Gene Research, College of Life Sciences, Peking University, Beijing, People’s Republic of China; 2 Peking-Tsinghua Center for Life Sciences, Peking University, Beijing, People’s Republic of China; 3 Key Laboratory of Photosynthesis and Environmental Molecular Physiology, Institute of Botany, the Chinese Academy of Sciences, Beijing, People’s Republic of China; 4 Department of Biology, University of Washington, Seattle, Washington, United States of America; 5 The National Plant Gene Research Center (Beijing), Beijing, People’s Republic of China; The University of North Carolina at Chapel Hill, UNITED STATES

## Abstract

The cuticle is a hydrophobic lipid layer covering the epidermal cells of terrestrial plants. Although many genes involved in *Arabidopsis* cuticle development have been identified, the transcriptional regulation of these genes is largely unknown. Previously, we demonstrated that AtCFL1 negatively regulates cuticle development by interacting with the HD-ZIP IV transcription factor HDG1. Here, we report that two bHLH transcription factors, AtCFL1 associated protein 1 (CFLAP1) and CFLAP2, are also involved in AtCFL1-mediated regulation of cuticle development. CFLAP1 and CFLAP2 interact with AtCFL1 both *in vitro* and *in vivo*. Overexpression of either *CFLAP1* or *CFLAP2* led to expressional changes of genes involved in fatty acids, cutin and wax biosynthesis pathways and caused multiple cuticle defective phenotypes such as organ fusion, breakage of the cuticle layer and decreased epicuticular wax crystal loading. Functional inactivation of *CFLAP1* and *CFLAP2* by chimeric repression technology caused opposite phenotypes to the *CFLAP1* overexpressor plants. Interestingly, we find that, similar to the transcription factor HDG1, the function of CFLAP1 in cuticle development is dependent on the presence of AtCFL1. Furthermore, both HDG1 and CFLAP1/2 interact with the same C-terminal C4 zinc finger domain of AtCFL1, a domain that is essential for AtCFL1 function. These results suggest that AtCFL1 may serve as a master regulator in the transcriptional regulation of cuticle development, and that CFLAP1 and CFLAP2 are involved in the AtCFL1-mediated regulation pathway, probably through competing with HDG1 to bind to AtCFL1.

## Author Summary

The cuticle is a continuous lipid layer covering the aerial parts of land plants. It is very important for the plants, especially for those in the drought area. The biosynthesis of cuticle have been studied well in past decades, however, the transcriptional regulation is still largely unknown. Here we found two new bHLH transcription factors, AtCFL1 associated protein 1 (CFLAP1) and its homolog CFLAP2, which could interact with AtCFL1, a previously identified negative regulator of *Arabidopsis* cuticle formation. Overexpression of *CFLAP1* and *CFLAP2* caused cuticle developmental defects, which are similar to the phenotypes of *AtCFL1* overexpression plants. Functional inactivation of CFLAP1 in *Arabidopsis* presents opposite phenotypes to those of its overexpressor. Interestingly, the function of CFLAP1 is dependent on the presence of AtCFL1. These results suggest that CFLAP1 and CFLAP2 regulate cuticle development by interacting with AtCFL1, and that AtCFL1 may work as a master regulator in the transcriptional regulation network.

## Introduction

All primary aerial surfaces of land plants are covered by a continuous hydrophobic layer, the cuticle, which is synthesized in the epidermal cells [1–4]. The cuticle layer has multiple functions, such as protecting plants against biotic and abiotic stresses, preventing postgenital organ fusion, and, as a crucial adaptive characteristic for terrestrial plants, preventing excessive non-stomatal water loss [3, 5]. The cuticle is mainly composed of cutin and waxes [3]. Cutin consists of C16 and C18 fatty acids cross-linked by ester bonds to form a porous three-dimensional net [6]. Waxes are mainly composed of very-long-chain fatty acids (VLCFAs) and their derivatives, such as aldehydes, alcohols, alkanes, ketones, and esters, with predominant carbon chain length ranging from C24 to C34 [3]. Waxes are embedded in the cutin polyester net and deposited on the aerial surface. The precursors for the biosynthesis of cutin and waxes are derived from C16 and C18 fatty acids, which are produced in the plastids via the *de novo* fatty acids biosynthesis pathway.

Although cutin is one of the most abundant lipid polymers in plants and forms the skeleton of the cuticle, its biosynthesis is not well understood. In *Arabidopsis*, glycerol-3-phosphate acyltransferases (GPATs), which catalyze the transfer of acyl groups from acyl-CoAs to glycerol-3-phosphate to form cutin monomers, were reported to play important roles in cutin biosynthesis. The cutin production in these loss-of-GPAT-function mutants was severely compromised [7–9]. The cutin monomers were polymerized to form cuticle by polymerases called cutin synthases. The cutin synthases, cutin deficient 1 (CD1) and Gly-Asp-Ser-Leu lipase 1 (GDSL1), were first identified in tomato [10, 11], and they seemed to have conserved function in land plants [12]. *BODYGUARD (BDG)*, a member of the α/β-hydrolase fold protein superfamily expressed in epidermal cells, is also involved in cuticle proper formation, since loss of *BDG* function resulted in cuticle defects and wax accumulation in rosette leaves [13]. Other proteins, such as cytochrome P450 monooxygenases, were also found important for the biosynthesis of cutin and very long fatty acid derivatives [14–17]. For instance, loss-of-function of the *LCR*, the gene encoding cytochrome P450 monooxygenase CYP86A8 that catalyzes ω-hydroxylation of fatty acids for the cross-linking of cutin monomers, caused cuticle defects and organ fusion in *Arabidopsis* [18].

In contrast to cutin biosynthesis, the biosynthesis of waxes in plants has been studied intensively in the past decade and the two major biosynthetic pathways, the acyl reduction pathway and the decarbonylation pathway, are well defined [1, 2, 4]. Many wax defective mutants have been identified in different plant species, such as *Arabidopsis eceriferum* (*cer*) mutants and maize *glossy* mutants [19–21]. Many of the *CER* genes encode enzymes involved in biosynthesis and derivatization of VLCFAs [22–28]. For example, *CER6/CUT1* encodes an enzyme required for the elongation of C24 VLCFAs [29–31]. In addition to the *CER* genes, some other genes encoding enzymes involved in the elongation and derivatization of VLCFAs have been characterized, such as *KCS1*, *KCS2/DAISY*, *KCS10/FDH*, *WAX2* and *KCS20* [32–39]. In addition, ATP-binding cassette (ABC) transporters are required for transport of VLCFAs and their derivatives [40–44].

The transcriptional regulation of the genes involved in cuticle formation has been the focus of research and some transcription factors have been reported recently. WIN1 (wax inducer 1), also known as SHN1, an AP2 domain containing transcription factor, was identified as a positive regulator of cuticle development, possibly by directly binding to the promoter of the long-chain acyl-CoA synthetase 2 (*LACS2*) gene and regulating its transcription [5, 45–47]. Furthermore, heterologous expression of two other AP2 transcription factors, WXP1 (wax production 1) and WXP2 from alfalfa (*Medicago sativa*), also led to wax accumulation in *Arabidopsis* leaves [48, 49], suggesting that AP2 transcription factors play important roles in wax biosynthesis. MYB proteins were also demonstrated to be involved in the regulation of cuticle development in *Arabidopsis* [50–52]. For instance, two MIXTA-like MYB transcription factors, MYB106 and MYB16, were recently found to regulate the transcription of *WIN1/SHN1* [53]. Furthermore, two homeodomain transcription factors, *i*.*e*., tomato CD2 (cutin deficient 2) and rice OCL1 (outer cell layer 1), were also reported to participate in the regulation of cuticle formation [54, 55]. In addition, other proteins, *e*.*g*., CER7 and CER9, could regulate cuticle development in the post-transcriptional level [56, 57]. Although several transcription factors and regulating proteins have been identified, what the transcriptional regulation networks of cuticle development are and how these transcription factors cooperate are far from clear.

We previously demonstrated that AtCFL1 played an important role in regulating cuticle development by interacting with a HD-ZIP IV transcription factor, HDG1, and modulating its function [58]. Here, we report the identification of two new AtCFL1-interacting proteins, AtCFL1 associated protein 1 (CFLAP1) and CFLAP2. CFLAP1 and CFLAP2 are bHLH transcription factors, the overexpression of which resulted in similar phenotypes to *AtCFL1* overexpressor plants. The *CFLAP1* overexpressor plants were defective in cuticle development in their rosette leaves and inflorescence stems. Inactivation of *CFLAP1* function by chimeric repression technology caused opposite phenotypes to the *CFLAP1* overexpressor plants. Interestingly, similar to HDG1, the proper function of CFLAP1 was AtCFL1-dependent. Furthermore, CFLAP1 and CFLAP2 interact with AtCFL1 through the C4 zinc finger domain at the C-terminus of AtCFL1, the same domain that HDG1 binds to. This C4 zinc finger domain is essential for AtCFL1 function. These results suggest that AtCFL1 may serve as the master regulator of cuticle development, and that CFLAP1 and CFLAP2 are involved in this AtCFL1-mediated regulation pathway, probably through competing with HDG1 to bind to AtCFL1.

## Results

### CFLAP1 Interacts with AtCFL1

**Fig 1:**
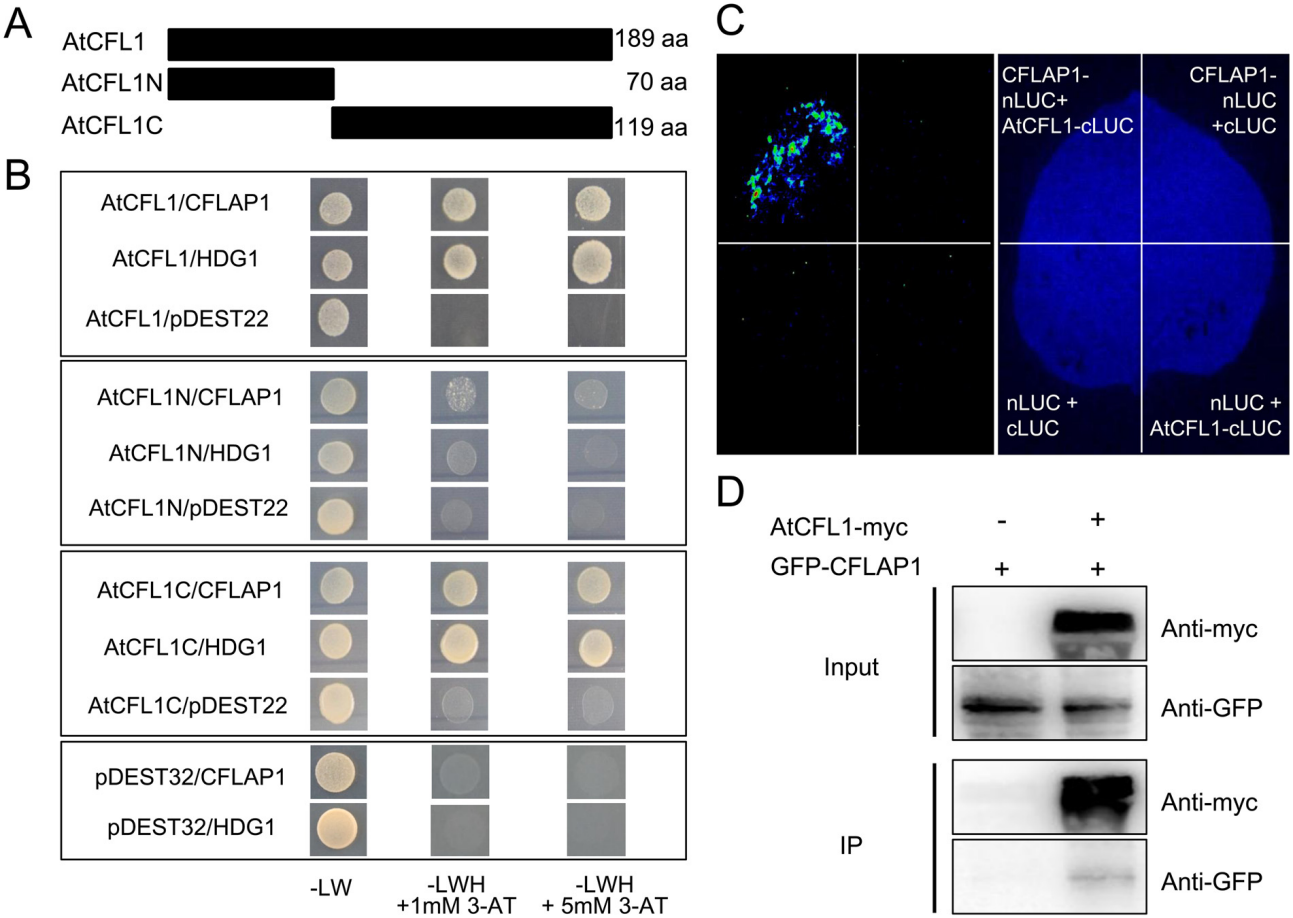
CFLAP1 interacts with AtCFL1 *in vitro* and *in vivo*. (A) Schematic diagram of AtCFL1 and its truncated proteins for yeast two-hybrid assay. AtCFL1, full length; AtCFL1N, N-terminal of AtCFL1 with 70 amino acid residues; AtCFL1C, C-terminal of AtCFL1 with 119 amino acid residues. (B) The results of yeast two-hybrid assay for the interactions of AtCFL1, AtCFL1N, and AtCFL1C with CFLAP1 respectively. The co-transformed yeast strains were plated on the control medium SD-Leu-Trp (SD-LW) and selective medium SD-Leu-Trp-His (SD-LWH) plus 3-amino-1, 2, 4-triazole (3-AT). HDG1 was used as a positive control and the empty plasmid pDEST22 as a negative control. The yeast co-transformed with empty plasmid pDEST32 together with HDG1 or CFLAP1 exhibited no auto-activation activities. (C) The result of the firefly luciferase complementation imaging assay. The CFLAP1-nLUC and cLUC-AtCFL1 were transiently expressed in the leaf of tobacco (*Nicotiana benthamiana*). CFLAP1-nLUC plus cLUC, nLUC plus cLUC-AtCFL1, and nLUC plus cLUC were used as three negative controls. (D) The immunoprecipitation assay. Lane 1, GFP-CFLAP1; Lane 2, co-expressed AtCFL1-myc and GFP-CFLAP1.

In our previous report, AtCFL1 is a negative regulator of cuticle development in *Arabidopsis*, since overexpression of *AtCFL1* caused serious cuticle-defective phenotypes. AtCFL1 could interact with and negatively modulate the function of a HD-ZIP transcription factor, HDG1. We found that 35S:*HDG1SRDX* plants, in which HDG1 was functionally inactivated by an SRDX repression motif fused to its C-terminus, had similar but weaker phenotypes to *AtCFL1* overexpressor plants [58], which suggests that there might be other factors involved in AtCFL1-mediated regulation of cuticle development. We used AtCFL1 as a bait to screen the *Arabidopsis* transcription factor library, which includes 1598 transcription factors [59], by yeast two-hybrid (YTH) assay. A putative basic helix-loop-helix (bHLH) transcription factor, encoded by *At1g51140*, later designated CFLAP1, was found to interact with AtCFL1. We made two truncated versions of AtCFL1, *i*.*e*., the N-terminal 70-aa region and the C-terminal 119-aa region (Fig 1A), and found that only the C-terminal region was able to interact with both HDG1 and CFLAP1 (Fig 1B), suggesting that the C-terminus of AtCFL1 is responsible for protein-protein interactions. The *in vivo* interaction between AtCFL1 and CFLAP1 was confirmed by the firefly luciferase complementation imaging assay (Fig 1C).

To further confirm the interaction between AtCFL1 and CFLAP1, we conducted a co-immunoprecipitation (co-IP) experiment in tobacco (*Nicotiana benthamiana*) leaves. We generated two constructs of the fusion proteins AtCFL1-myc and GFP-CFLAP1 (S1 Fig) and transiently co-expressed them in tobacco leaves. The results showed that GFP-CFLAP1 was co-immunoprecipitated with AtCFL1-myc (Fig 1D). These results demonstrate that CFLAP1 indeed interacts with AtCFL1.

### Overexpression of *CFLAP1* Caused Cuticle Defects

**Fig 2:**
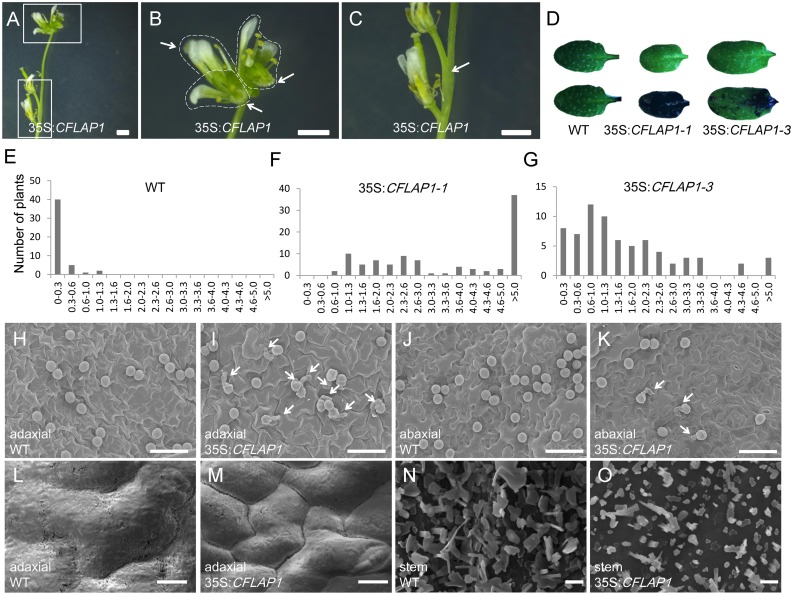
Overexpression of *CFLAP1* caused cuticle defective phenotypes. (A) Organ-fusion in 35S:*CFLAP1* plants. (B) and (C), higher magnification of boxed regions in (A). Dashed lines indicate the fused flowers in (B); arrowheads indicate the styles of the fused flowers in (B) and a fused pedicel in (C). Bar = 1 mm. (D) TB staining of the rosette leaves. Top, before TB staining; bottom, after TB staining for 2 minutes. From the left to right, wild type, 35S:*CFLAP1-1* and 35S:*CFLAP1-3*, respectively. (E) to (G) Quantificational analysis on the intensity of TB staining. The number of horizontal axis is A_630_:A_435_ ratio (a quantification of TB staining intensity, the higher the ratio, the higher intensity of TB absorption). From (H) to (J), wild type, 35S:*CFLAP1-1*, and 35S:*CFLAP1-3*, respectively. (H) to (K) The results of pollen germination assay. (H) and (I), adaxial surfaces of wild type and 35S:*CFLAP1*; (J) and (K), abaxial surfaces of wild type and 35S:*CFLAP1*; arrowheads indicate germinated pollen gains. Bar = 50 μm. (L) and (M) Cryo-SEM images of adaxial surface of wild-type and 35S:*CFLAP1* rosette leaves. Bar = 10 μm. (N) and (O) SEM images of the epicuticular wax crystals on the stem of wild type and 35S:*CFLAP1*. Bar = 2 μm.

CFLAP1, also designated FBH3, and its three homologs work as transcriptional activators by binding to the E-box *cis*-element in the *CONSTANS* (*CO*) promoter to regulate flowering in *Arabidopsis* [60]. To investigate the AtCFL1-associated function of CFLAP1, we first overexpressed *CFLAP1* in wild-type *Arabidopsis*. The T2 progenies of *CFLAP1* overexpressor plants exhibited multiple phenotypes, such as early flowering, dwarfism, rough siliques (S2A to S2D Fig), and in particular, postgenital organ fusion (Fig 2A to 2C), similar to *AtCFL1* overexpressor plants. To test whether cuticle development was abnormal in *CFLAP1* overexpressor plants, we performed toluidine-blue (TB) staining assay [61] on the fifth leaves of two independent transgenic lines, 35S:*CFLAP1-1* and 35S:*CFLAP1-3*. We found that the leaves of both transgenic plants were stained by TB, suggesting that overexpression of *CFLAP1* caused cuticle defects in *Arabidopsis* (Fig 2D). To quantify the TB staining, we measured the A_630_:A_435_ ratio of the leaves. The A_630_:A_435_ ratio, in which TB absorbance is at A_630_ and plant material absorbance is at A_435_, represents the relative amount of bound TB dye. We found that the expression level of *CFLAP1* was positively correlated with the severity of the TB staining phenotypes in the *CFLAP1* overexpressor plants (Fig 2E to 2G and S2E Fig). These results suggest that over-expression of *CFLAP1* affects cuticle development in *Arabidopsis*.

To confirm the cuticle defective phenotypes in 35S:*CFLAP1* plants, we performed a pollen germination assay. The rationale was that fragmentation of the hydrophobic layer of the plant leaves, due to cuticle defects, would nullify the prohibition of pollen germination on the leaf surfaces [13, 18, 32, 62, 63]. The results showed that, whereas only about 0.62% of pollen grains germinated on the leaf surfaces of wild-type plants (Fig 2H and 2J), 44.16% and 19.65% of pollen grains germinated on the adaxial and abaxial leaf surfaces of 35S:*CFLAP1* plants, respectively (Fig 2I and 2K and Table 1). This suggests that cuticle development is indeed compromised in *CFLAP1* overexpressor plants.

**Table 1:** Rate of pollen germination on rosette leaf surfaces.

Genotype	Leaf surface	Number of pollen grains counted	Number of pollen grains germinated	Germination rate %
Wild type	Adaxial	1764	11	0.62
35S:*CFLAP1*	Adaxial	1413	624	44.16 ***
Wild type	Abaxial	1454	9	0.62
35S:*CFLAP1*	Abaxial	982	193	19.65 ***

To further investigate the cuticle development defects in *CFLAP1* overexpressor plants, we conducted scanning electron microscopy (SEM) analysis on rosette leaves and inflorescence stems. We found that the epicuticular wax crystals covering the epidermal surface of the leaf were changed in the 35S:*CFLAP1* plants. The gap between the two epidermal cells was larger than that in the wild type (Fig 2L and 2M). Prominently, the crystals in the stems of 35S:*CFLAP1* plants are less and smaller than those of wild type (Fig 2N and 2O), suggesting that the cuticle development in this transgenic line might be compromised. Meanwhile, transmission electron microscope (TEM) analysis revealed that the continuity of cutin layer was slightly affected, and the density of cutin was decreased in 35S:*CFLAP1* plants (S2F and S2G Fig). These results suggest that cuticle development is affected in the *CFLAP1* overexpressor plants, and that CFLAP1, similar to AtCFL1, negatively regulates cuticle development in *Arabidopsis*.

### Epicuticular Wax Components Were Changed in 35S:*CFLAP1* Plants

**Fig 3:**
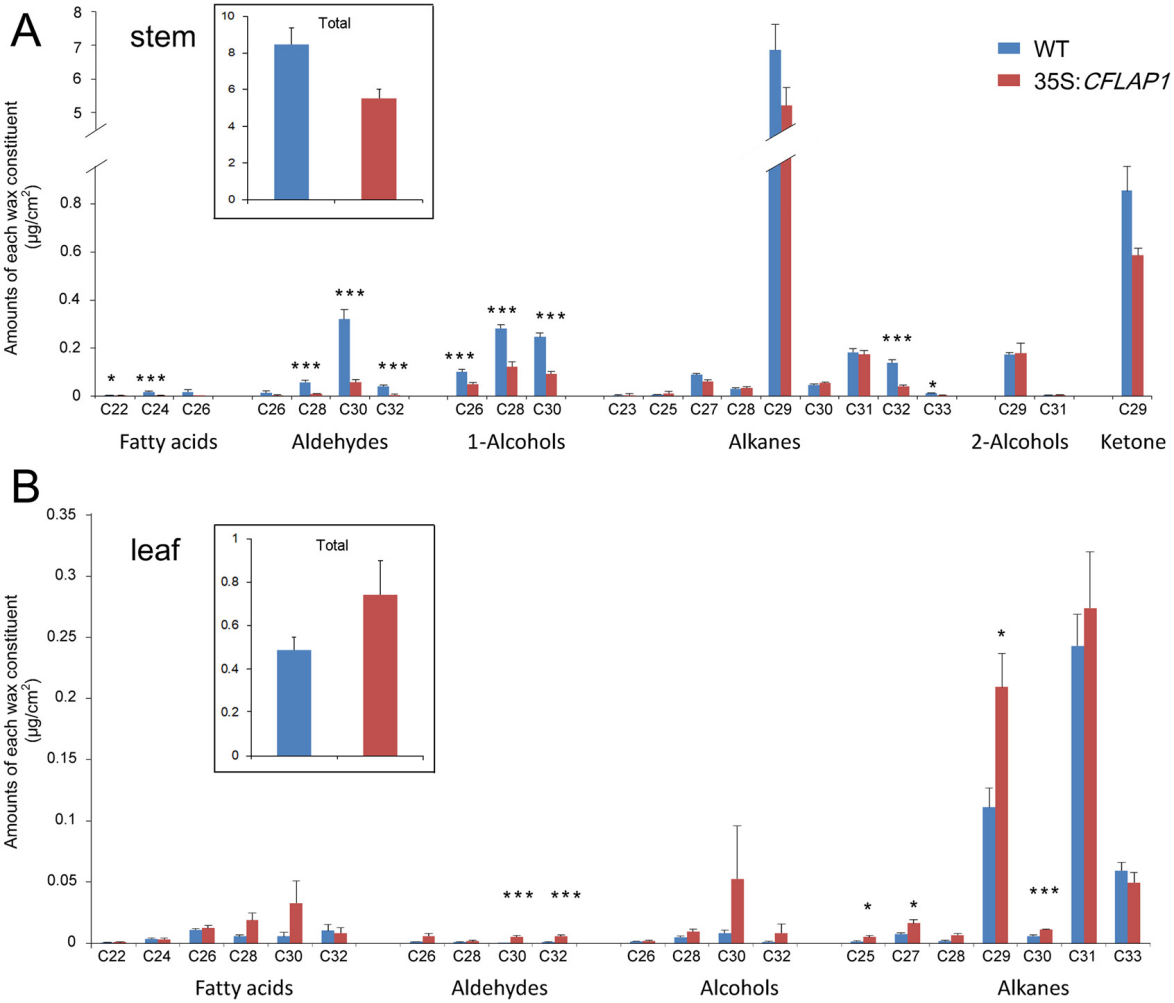
Epicuticular wax components analysis in 35S:*CFLAP1* and wild-type plants. (A) Epicuticular wax components in 35S:*CFLAP1* and wild-type stems. Numbers indicate the main chain length of each constituent. Each value is the mean + SD of three biological replicates. At least 5 individual stems were used for each replicate. (B) Epicuticular wax components of 35S:*CFLAP1* and wild-type rosette leaves. Numbers indicate the main chain length of each constituent. Each value is the mean + SD of three biological replicates. At least 7 individual rosette leaves were used for each replicate. Level of significance obtained with a Student’s *t* test is marked by the following: *, p<0.05; ***, p<0.01.

To further investigate how cuticle development was affected in *CFLAP1* overexpressor plants, we analyzed the epicuticular wax composition by gas chromatography-mass spectrometry (GC-MS). The results showed that the total amount of waxes in the inflorescence stems of 35S:*CFLAP1* plants was reduced to two-thirds of the wild type (5.50 ± 0.51 μg/cm^2^ vs. 8.47 ± 0.88 μg/cm^2^, Fig 3A), consistent with the phenotype of decreased crystal observed by SEM. The C24-and-above components, such as C24 fatty acids, C28, C30 and C32 aldehydes, C26, C28 and C30 alcohols, and C32 alkanes, were significantly reduced in the *CFLAP1* overexpressor plants (Fig 3A). The epicuticular waxes, however, were increased in the leaves of 35S:*CFLAP1* plants (Fig 3B). For instance, the amounts of C30 and C32 aldehydes, and C30 alkane were increased by 13.9-, 6.8- and 18.9-fold, respectively (Fig 3B). The C25, C27 and C29 alkanes were also obviously increased. This was similar to the observations of other cuticle-defective mutants, *fdh* and *lcr* [18, 32, 64]. These data indicate that the epicuticular wax composition is altered by *CFLAP1* overexpression.

### CFLAP2, A Homolog of CFLAP1, Also Interacts with AtCLF1

**Fig 4:**
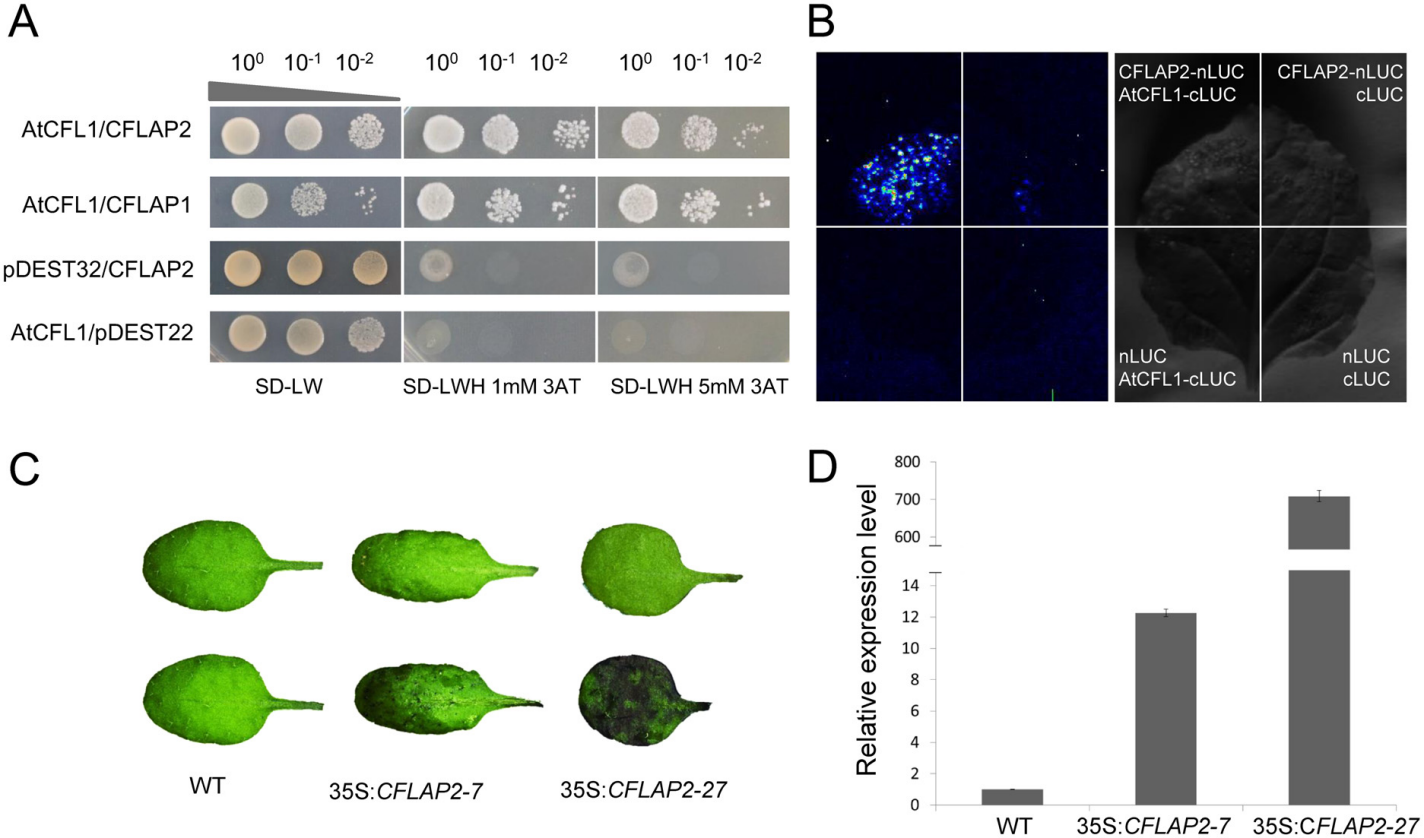
AtCFL1 interacts with CFLAP2 and the plants overexpressing *CFLAP2* had cuticle defective phenotypes. (A) The result of yeast two-hybrid assay for the interaction of CFLAP2/FBH1 with AtCFL1. The co-transformed yeast strains were plated on the control medium SD-LW and selective medium SD-LWH plus 3-AT. AtCFL1/CFLAP1 was used as a positive control, and the empty plasmid pDEST32/CFLAP2 and AtCFL1/ empty plasmid pDEST22 were used as negative controls. (B) The result of the firefly luciferase complementation imaging assay. The CFLAP2-nLUC and cLUC-AtCFL1 were transiently expressed in the leaf of tobacco. CFLAP2-nLUC plus cLUC, nLUC plus cLUC-AtCFL1 and nLUC plus cLUC were used as three negative controls. (C) TB staining of rosette leaf. Top, before TB staining; bottom, after TB staining. From the left to right, wild type, 35S:*CFLAP2-7* and 35S:*CFLAP2-27*, respectively. (D) Relative expression level of *CFLAP2*. The expression level in wild types is set to 1.0. The error bars represent the SD of three biological replicates.

There are three homolog genes of *CFLAP1/FBH3* in *Arabidopsis* [60]. We found that, in addition to CFLAP1/FBH3, another homolog FBH1, but not FBH2 or FBH4, also interacted with AtCFL1 (Fig 4A and S3A Fig). We designated FBH1 as CFLAP2. The firefly luciferase complementation imaging assay confirmed the *in vivo* interaction between CFLAP2 and AtCFL1 in tobacco leaves (Fig 4B). Overexpression of *CFLAP2* in *Arabidopsis* produced cuticle defective phenotypes similar to those in 35S:*CFLAP1* plants (Fig 4C). TB staining analysis on two independent lines, 35S:*CFLAP2-7* and 35S:*CFLAP2-27*, showed that the staining intensity in 35S:*CFLAP2-27* leaves was higher than in 35S:*CFLAP2-7* leaves (Fig 4C), and was positively correlated with the expression levels of *CFLAP2* in these two lines (Fig 4D). In comparison, over-expression of either *FBH2* or *FBH4* did not produce a cuticle defective phenotype (S3B to S3F Fig). These data suggest that CFLAP1 and CFLAP2 have overlapping functions in regulating cuticle development, most likely through interacting with AtCFL1.

### Chimeric Repression of *CFLAP1* Produced Opposite Phenotypes to Those of 35S:*CFLAP1* Plants

**Fig 5:**
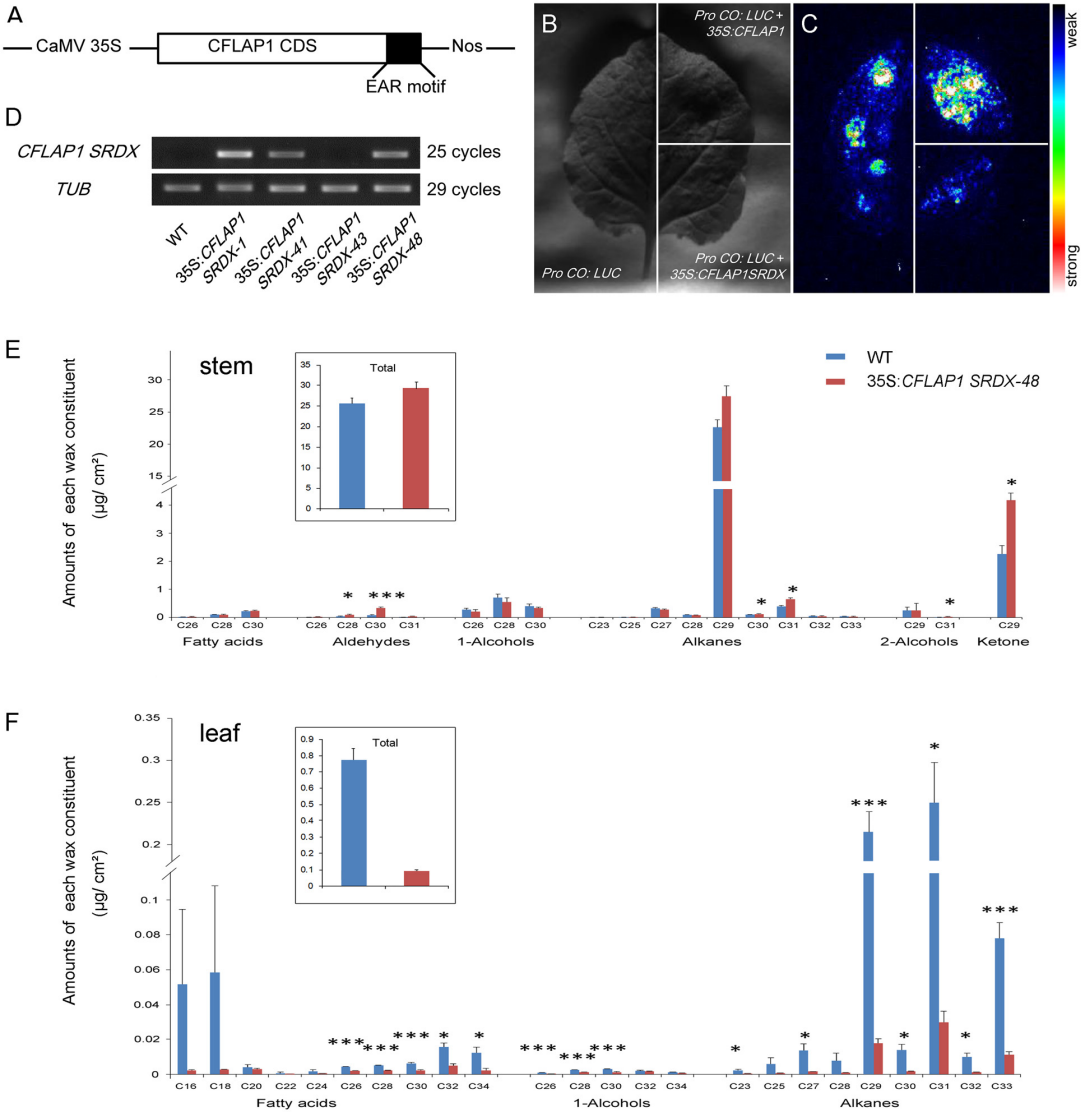
35S:*CFLAP1SRDX* plants had opposite phenotypes to 35S:*CFLAP1* plants. (A) The schematic diagram of 35S:*CFLAP1SRDX* construct. CaMV35S, CFLAP1 CDS, EAR motif and Nos represent the 35S promoter, *CFLAP1* coding sequence, the repression domain of 12 amino acid residues, and the *NOS* terminator sequence, respectively. (B) and (C) Transient effector-reporter expression assay in tobacco leaves, with the *CO* promoter driving a luciferase gene as a reporter, and the 35S:*CFLAP1* and 35S:*CFLAP1SRDX* as effectors. Luminescence imaging is shown 48 hours after co-infiltration with the constructs indicated at left. (D) The result of RT-PCR. From right to left, wild type, transgenic lines 35S:*CFLAP1SRDX-1*, 35S:*CFLAP1SRDX-41*, 35S:*CFLAP1SRDX-43*, and 35S:*CFLAP1SRDX-48*, respectively.*TUB2* was used as an internal control. (E) Epicuticular wax components of 35S: *CFLAP1SRDX-48* and wild-type stems. Numbers indicate the main chain length of each constituent. Each value is the mean + SD of three biological replicates. At least 5 individual stems were used for each replicate. (F) Epicuticular wax components of 35S: *CFLAP1SRDX-48* and wild-type rosette leaves. Numbers indicate the main chain length of each constituent. Each value is the mean + SD of three biological replicates. At least 7 individual rosette leaves were used for each replicate. Level of significance obtained with a Student’s *t* test is marked by the following: *, p<0.05; ***, p<0.01.

The single loss-of-function mutant *cflap1* (SALK_049022c) had no obvious phenotype in cuticle development or flowering time, consistent with the previous report [60]. We also analyzed the epicuticular wax compositions of the previously reported quadruple mutant *fbh1 fbh2 fbh3 fbh4* [60] by GC-MS. The results showed that, in the inflorescence stems, the total amount of waxes was increased in the quadruple mutant compared to that in wild type, especially for the amount of several alkanes including C30, C31, C32 and C33, which were significantly accumulated, whereas in the rosette leaves, however, the amount of wax was decreased, especially for the amount of C32 and C34 fatty acids (S4 Fig). These wax compositions were slightly opposite to those in *35S*:*CFLAP1* plants (Fig 3). The phenotype of this *fbh1 fbh2 fbh3 fbh4* quadruple mutant was also relatively weak (*i*.*e*., slightly later flowering [60]), possibly due to incomplete knock-out of the three *CFLAP1*-homologous genes in the quadruple mutant (S4 Fig). Therefore, to elucidate the function of *CFLAP1* and its homologous genes, we used chimeric repressor technology [65–67] to further knock down the activities of CFLAP1 and its homologs by overexpressing an ethylene response factor (ERF)-associated amphiphilic repression (EAR) motif-fused *CFLAP1* construct, 35S:*CFLAP1SRDX*, in wild-type *Arabidopsis* (Fig 5A).

Because CFLAP1/FBH3 was reported to directly bind to the promoter of *CO* to control flowering time [60], we used the *CO* promoter to drive the luciferase (*LUC*) reporter gene to test whether the CFLAP1 activity is compromised by the 35S:*CFLAP1SRDX* construct *in vivo*. The result showed that co-filtration of *ProCO*:*LUC* and 35S:*CFLAP1* into tobacco leaves generated much stronger luminescence signal than infiltration with *ProCO*:*LUC* alone (Fig 5B and 5C), suggesting that CFLAP1 activates *CO* in tobacco, consistent with the previous report [54]. However, when *ProCO*:*LUC* and 35S:*CFLAP1SRDX* were co-infiltrated, the luminescence signal was significantly reduced (Fig 5B and 5C). These results suggest 35S:*CFLAP1SRDX* greatly represses CFLAP1 function *in vivo*.

Fifty 35S:*CFLAP1SRDX* independent transgenic lines were obtained and the expression level of the modified *CFLAP1* was examined in four lines (Fig 5D), among which the 35S:*CFLAP1SRDX-48* was used for further analysis. The T2 progeny of 35S:*CFLAP1SRDX-48* was apparently late-flowering (S5A and S5B Fig). We investigated the cuticle composition of these plants by GC-MS. In contrast to the *CFLAP1* overexpressor plants, we found that the inflorescence stems of 35S:*CFLAP1SRDX-48* had 29.38 ± 1.50 μg/cm^2^ epicuticular wax, a slight (not significant) increase compared with the wild type (25.5 ± 1.41 μg/cm^2^, Fig 5E), although crystal loading was no obviously increased on the stem of the transgenic plants compared with wild type under SEM observation (S5C and S5D Fig). Furthermore, the C28 and C30 aldehydes, C30 and C31 alkanes, C31 alcohols and C29 ketones were significantly increased (Fig 5E). Interestingly, the wax in the leaves of 35S:*CFLAP1SRDX-48* plants was reduced compared with the wild type (Fig 5F), which was exactly the opposite of 35S:*CFLAP1* plants. Similar change trends were observed in another independent transgenic line (S6 Fig). These results indicated that CFLAP1 negatively regulates cuticle development in *Arabidopsis*.

### High-Throughput Transcriptome Analysis Revealed Significant Changes in Expression of Lipid Related Genes in 35S:*CFLAP1* and 35S:*CFLAP1SRDX* Plants

**Fig 6:**
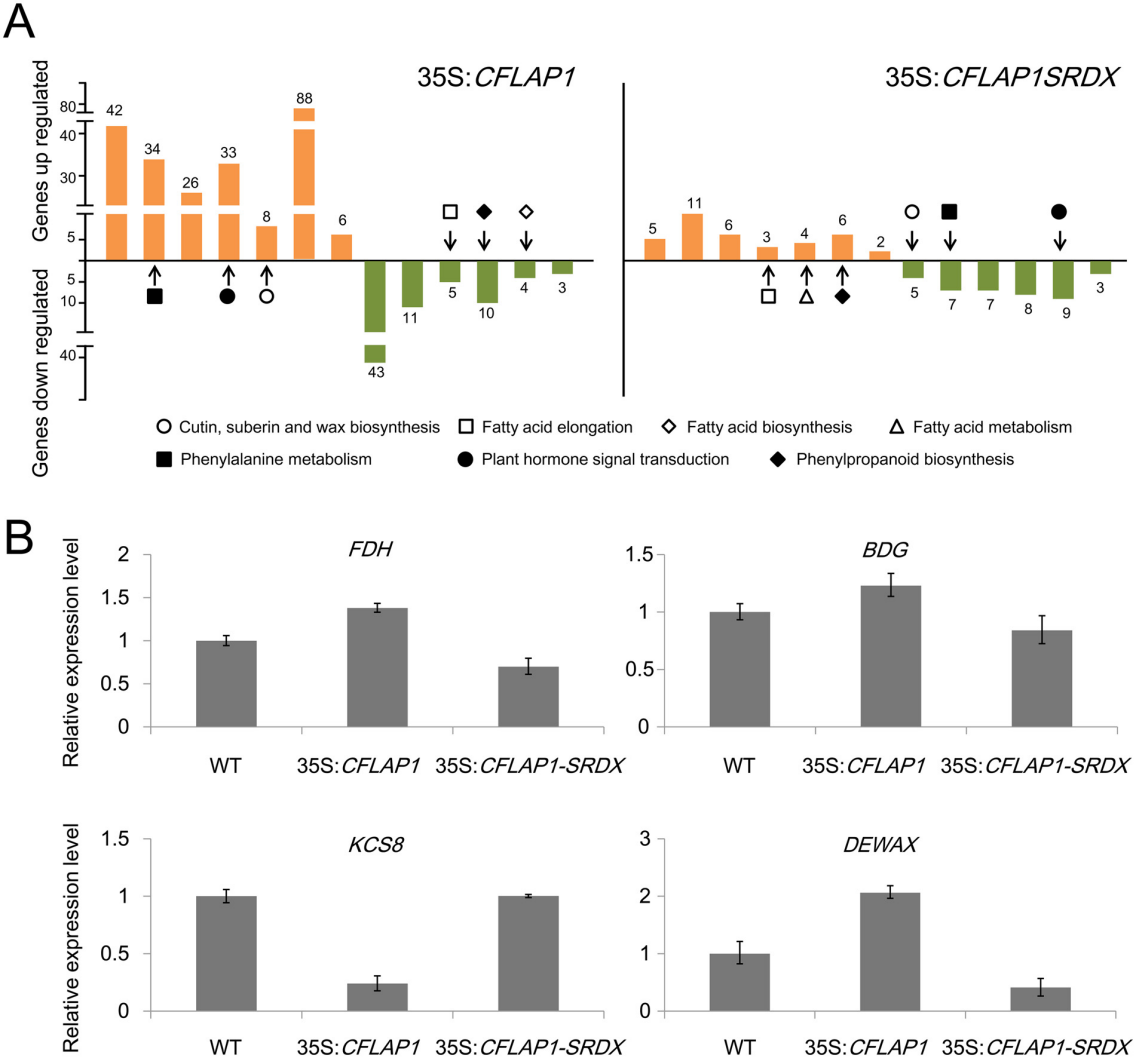
Lipid-related pathways were oppositely regulated in 35S:*CFLAP1* plants and 35S:*CFLAP1SRDX* plants. (A) The orange columns indicate the numbers of the up-regulated genes mapped to the enriched pathways. The green columns indicate the numbers of the down-regulated genes mapped to the enriched pathways. Detailed information was further shown in Tables 2 and 3. Arrows highlight similar pathways with opposite expression trends in 35S:*CFLAP1* plants and 35S:*CFLAP1SRDX* plants. (B) Relative expression levels of *FDH*, *BDG*, *KCS8* and *DEWAX* in wild type, 35S:*CFLAP1* and 35S:*CFLAP1-SRDX* plants. The expression level in wild type is set to 1.0. The error bars represent the SD of three biological replicates.

To investigate the CFLAP1-affected genes in the *CFLAP1* overexpressor (35S:*CFLAP1*) and suppressor (35S:*CFLAP1SRDX*) plants, we adopted the high-throughput RNA sequencing technology to compare the transcriptome profiles of either the 35S:*CFLAP1* or 35S:*CFLAP1SRDX* plants with wild type (S1 and S2 Tables). In the 35S:*CFLAP1* plants, 2576 genes were up-regulated and 1505 genes down-regulated by twofold and above. In the 35S:*CFLAP1SRDX* plants, 748 up-regulated genes and 862 down-regulated genes were identified. KOBAS pathway analysis [68] showed that, in the 35S:*CFLAP1* plants, the genes involved in cutin, suberin and wax biosynthesis were enriched in the up-regulated genes (Fig 6A and Table 2), while the genes involved in fatty acid elongation and fatty acid biosynthesis were enriched in the down-regulated genes (Fig 6A and Table 3). Interestingly, in the 35S:*CFLAP1SRDX* plants, we found that the genes enriched in up-regulated category were involved in fatty acids elongation and metabolism genes (Fig 6A and Table 2), while the genes enriched in the down-regulated category were involved in cutin, suberin and wax biosynthesis genes (Fig 6A and Table 3), opposite to those in 35S:*CFLAP1* plants. These results suggest that CFLAP1 participates cuticle development through specifically regulating the transcription of those genes involved in lipid-related metabolism.

**Table 2:** KEGG pathways in which genes up-regulated by more than 2-fold in these transgenic plants.

Plants	Term	P-value
35S:*CFLAP1*	Phenylpropanoid biosynthesis	0
	Phenylalanine metabolism	4.219E-15
	Plant-pathogen interaction	1.478E-4
	Plant hormone signal transduction	0.001
	Cyanoamino acid metabolism	0.001
	Biosynthesis of secondary metabolites	0.002
	Cutin, suberin and wax biosynthesis	0.004
	Tryptophan metabolism	0.007
	Glucosinolate biosynthesis	0.016
	Starch and sucrose metabolism	0.016
	Zeatin biosynthesis	0.030
	ABC transporters	0.042
35S:*CFLAP1SRDX*	Tryptophan metabolism	0.0006
	Plant hormone signal transduction	0.0017
	Phenylalanine metabolism	0.006
	Fatty acid elongation	0.011
	Fatty acid metabolism	0.021
	Phenylpropanoid biosynthesis	0.022
	Glucosinolate biosynthesis	0.033

**Table 3:** KEGG pathways in which genes down-regulated by more than 2-fold in these transgenic plants.

Plants	Term	P-value
35S:*CFLAP1*	Ribosome	6.766E-11
	Ribosome biogenesis in eukaryotes	9.836E-4
	Fatty acid elongation	0.004
	Phenylpropanoid biosynthesis	0.012
	Fatty acid biosynthesis	0.024
	Lysine biosynthesis	0.029
35S:*CFLAP1SRDX*	Cutin, suberin and wax biosynthesis	0.0007
	Phenylalanine metabolism	0.0024
	Phenylpropanoid biosynthesis	0.011
	Protein processing in endoplasmic reticulum	0.023
	Plant hormone signal transduction	0.027
	Alanine, aspartate and glutamate metabolism	0.042

We further adopted qRT-PCR to confirm the RNA-seq results. We found that, in 35S:*CFLAP1* plants, the transcription levels of several key genes involved in cuticle development were disturbed, among which *FDH*, *BDG* and *DEWAX* [69] were up-regulated and *KCS8* was down-regulated. Meanwhile, the changes of these gene expressions exhibited opposite patterns to those in the 35S:*CFLAP1-SRDX* plants (Fig 6B), consistent with the phenotypic changes in 35S:*CFLAP1* and 35S:*CFLAP1-SRDX* plants.

### Regulation of Cuticle Development by CFLAP1 Is AtCFL1-Dependent

**Fig 7:**
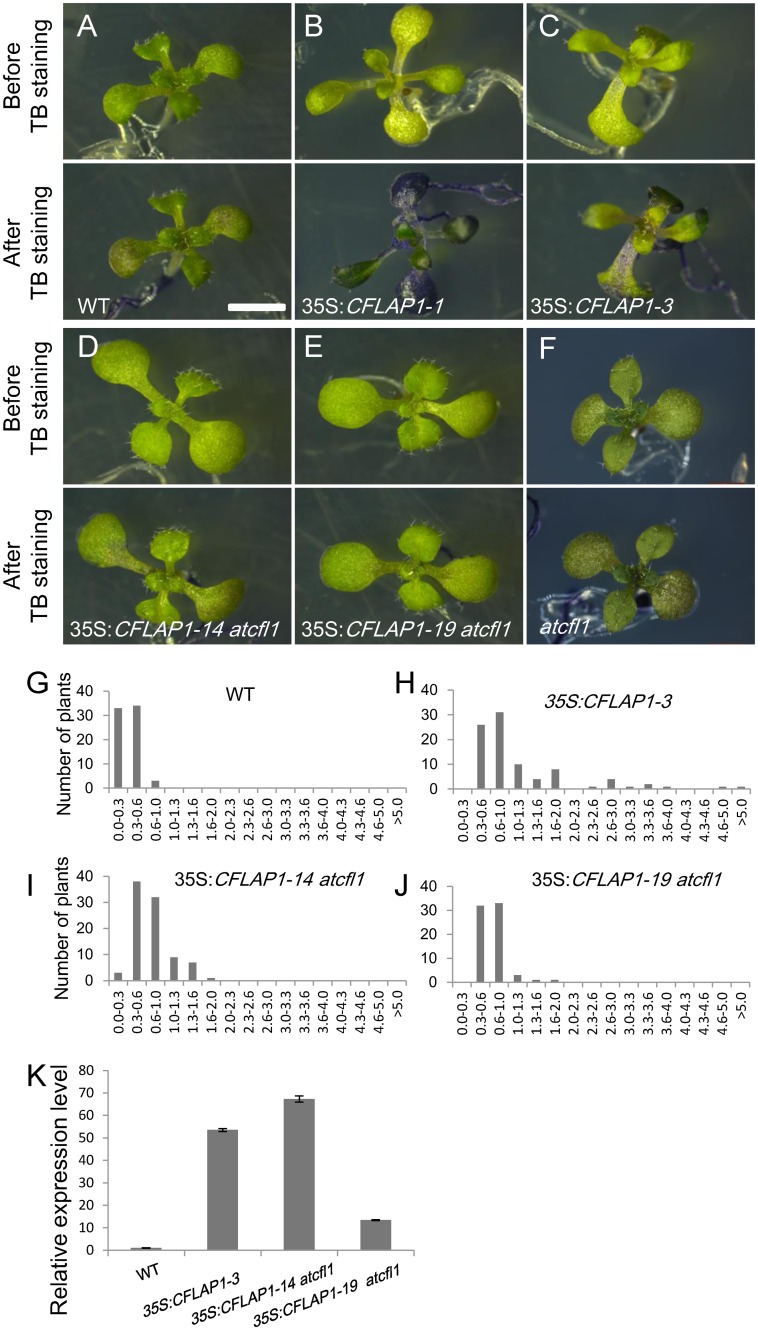
TB staining assay on the plants overexpressing *CFLAP1* in the *atcfl1* background. (A) to (F) TB staining assay of 14-day-old seedlings. (A), wild type; (B), 35S: *CFLAP1-1*; (C), 35S:*CFLAP1-3*; (D) and (E), 35S:*CFLAP1-14* and 35S:*CFLAP1-19* in the background of *atcfl1* mutant respectively, (F), *atcfl1* mutant. Top, before TB staining; bottom, after TB staining. Bar = 2 mm. (G) to (J) Quantification of the TB staining intensity, the number of horizontal axis is A_630_:A_435_ ratio. (G) wild type, (H) 35S:*CFLAP1-3*, (I) and (J) 35S:*CFLAP1-14* and 35S:*CFLAP1-19* in the background of *atcfl1* mutant respectively. (K) Relative expression level of *CFLAP1* in the wild type, 35S:*CFLAP1-3*, 35S:*CFLAP1-14* and 35S:*CFLAP1-19* in the background of *atcfl1* mutant. The expression level in the wild type is set to 1.0, and error bars represent the SD of three biological replicates.

To clarify the genetic relationship between *AtCFL1* and *CFLAP1*, we overexpressed *CFLAP1* in the loss-of-function mutant *atcfl1* background. In the TB staining assay, the staining intensity of 35S:*CFLAP1* leaves in the *atcfl1* mutant background was dramatically decreased compared with those in the wild type background (Fig 7A to 7F), even when the expression level of *CFLAP1* was comparable to that in the 35S:*CFLAP1-3* plant (Fig 7K). While the A_630_:A_435_ ratio ranged from 0.3 to 5.0 in 35S:*CFLAP1-3* compared with a ratio below 1.0 in the wild type, the ratio of 35:*CFLAP1-14* and 35:*CFLAP1-19* in the *atcfl1* mutant background was significantly decreased with most plants below 1.0, which was close to the wild type ratio (Fig 7G to 7J). These results suggested that AtCFL1 and CFLAP1 worked in the same genetic pathway to regulate cuticle development, and that CFLAP1 worked in an AtCFL1-dependent manner.

### The Expression Patterns of *CFLAP1* and *CFLAP2* Were Partially Overlapped with That of *AtCFL1*

**Fig 8:**
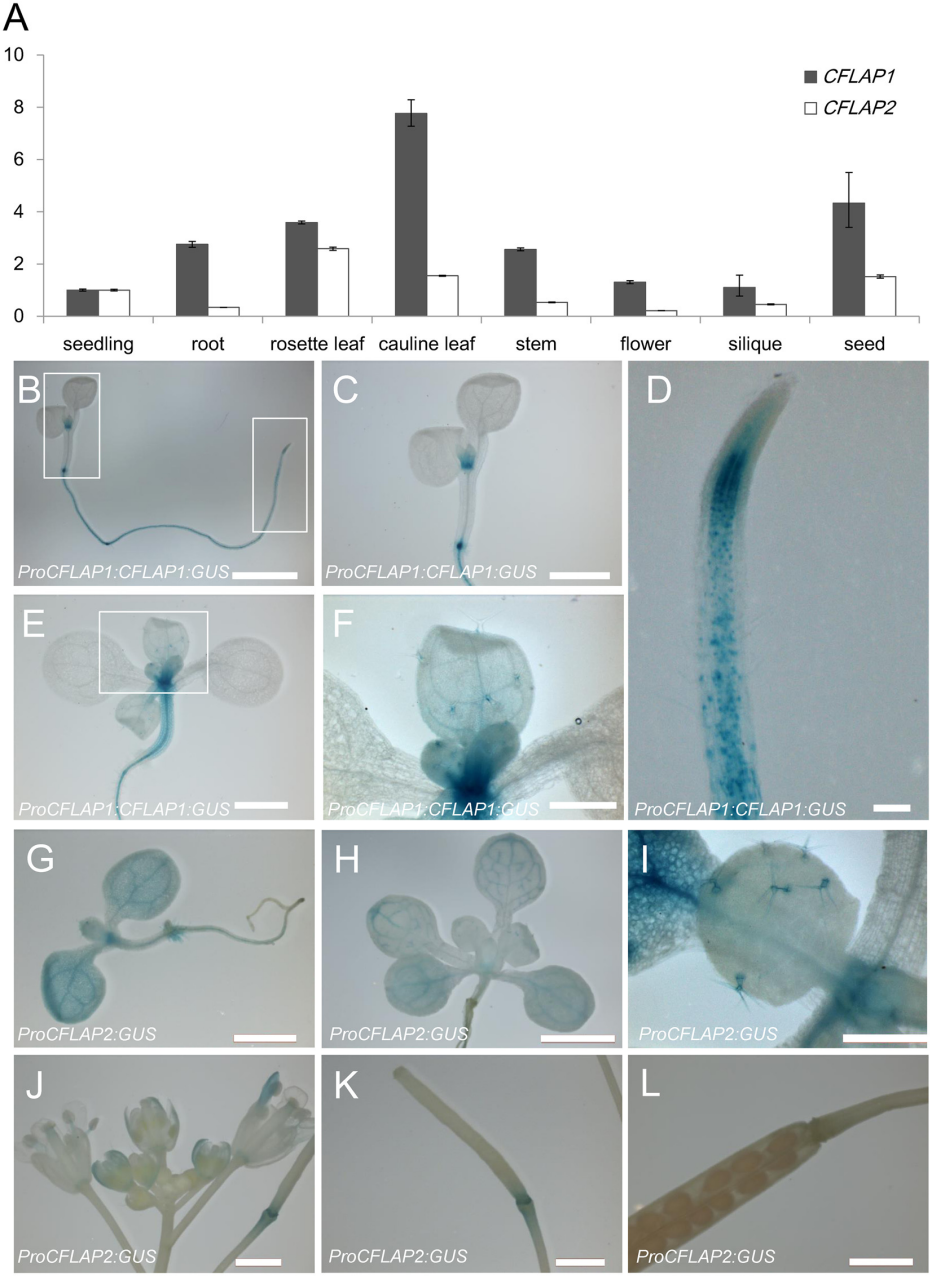
The expression patterns of *CFLAP1* and *CFLAP2*. (A) Analysis of CFLAP1/2 expression levels in different organs of wild-type *Arabidopsis* by qRT-PCR. The expression level of seedling is set to 1.0, and error bars represent the SD of three biological replicates. (B) to (F) The expression pattern of CFLAP1 in *Arabidopsis*. (B) GUS-stained one-week-old seedling. (C) and (D) Higher magnification of boxed regions in (B). (E) GUS-stained two-week-old seedling. (F) Higher magnification of boxed region in (E). (G) to (L) The expression pattern of CFLAP2 in *Arabidopsis*. (G) GUS-stained one-week-old seedling. (H) GUS-stained two-week-old seedling. (I) GUS-stained trichomes in true leaves of two-week-old seedling. (J) GUS-stained inflorescence stem with buds, flowers, and siliques. (K) GUS-stained young silique. (L) GUS-stained the abscission zone of mature silique. Bars = 2 mm in (B) and (H), 1 mm in (C), (E), (G), (J), (K) and (L), 500 μm in (F) and (I), 100 μm in (D).

To investigate the gene expression patterns of *CFLAP1* and *CFLAP2*, we first analyzed the expression level of these two genes in wild type using qRT-PCR. The transcripts of *CFLAP1* and *CFLAP2* were ubiquitously detected in 14-day-old seedlings and in all the tissues of mature plants (Fig 8A). We then fused a 4.5-kb fragment including genomic sequence of *CFLAP1* to *Escherichia coli* β-glucuronidase (GUS) reporter gene and transformed it into wild-type *Arabidopsis*. The GUS activities were observed in root, young leaf and trichomes (Fig 8B to 8F). Furthermore, the GUS signal displayed a spotted pattern in the root tip (Fig 8D), suggesting that CFLAP1 protein localizes in the nuclei, consistent with the observation of 35S:*GFP-CFLAP1* plant (S7 Fig) and the previous report [70]. In the *ProCFLAP2*:GUS transgenic plants, the GUS activity was observed in cotyledon, leaf veins, trichomes and flowers (Fig 8G to 8J). Strong GUS signal was also observed in the abscission zone at the bottom of young siliques (Fig 8K), but not in the mature siliques (Fig 8L). Since *AtCFL1* is reported expressed in the trichomes, guard cells, root endodermis and stigmatic papillar cells [58], our results suggest that the expression patterns of *CFLAP1* and *CFLAP2* are partially overlapped with that of *AtCFL1*, providing the base for the interaction of AtCFL1 with CFLAP1/2.

### A Putative Zinc Finger Domain in the C-Terminus of AtCFL1 Is Necessary for the Interactions between AtCFL1 and Transcription Factors

**Fig 9:**
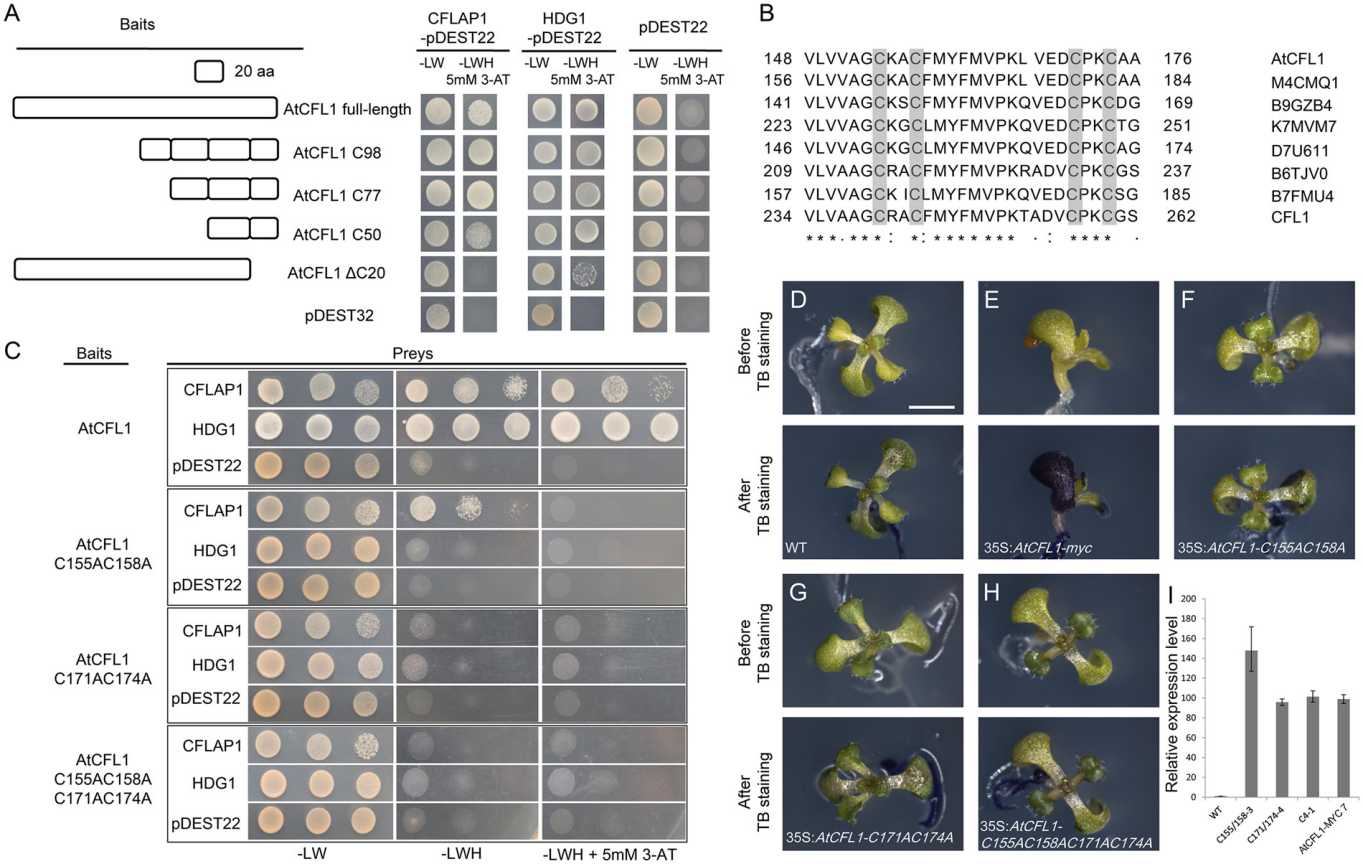
The C-terminal putative zinc finger domain of AtCFL1 is critical for both its interactions with transcription factors and its proper function. (A) The schematic diagram of AtCFL1 and its truncated proteins for yeast two-hybrid assay. From top to bottom, the full length, 98 amino acid residues(C98), 77 amino acid residues(C77) and 50 amino acid residues(C50) of AtCFL1, and C-terminal 20 amino acid residues deleted (ΔC20) respectively. The empty pDEST32 was used as a negative control. The co-transformed yeast strains were plated on the control medium SD-LW and selective medium SD-LWH plus 3-AT. The empty pDEST22 was used as a negative control. (B) Alignment of the C-terminal amino acid residues of AtCFL1, the orthologous proteins from *Brassica rapa subsp*. *Pekinensis* (M4CMQ1), *Populus trichocarpa* (B9GZB4), *Glycine max* (K7MVM7), *Vitis vinifera* (D7U611), *Zea mays* (B6TJV0), *Humulus japonicas* (B7FMU4) and rice (CFL1). Asterisks indicate identical residues, colons indicate highly similar residues and dots indicate slightly similar residues, shades indicate the four cysteine residues of the putative C4 zinc finger. (C) The results of yeast two-hybrid assay for the interactions of CFLAP1 and HDG1 with mutated AtCFL1s. The baits were wild-type AtCFL1, AtCFL1 with C155 and C158 residues mutated, AtCFL1 with C171 and C174 residues mutated and AtCFL1 with C155, C158, C171 and C174 residues mutated respectively. The co-transformed yeast strains were plated on the control medium SD-LW and selective medium SD-LWH plus 3-AT. The empty pDEST22 was used as a negative control. (D) to (H) TB staining assay of 14-day-old seedlings. (D), wild type; (E), 35S:*AtCFL1-myc*; (F), 35S:*AtCFL1-C155AC158A*; (G), 35S:*ATCFL1-C171AC174A*; (H) 35S:*ATCFL1-C155AC158A C171AC174A*. Top, before TB staining; bottom, after TB staining. Bar = 1 mm. (I) The relative expression levels of *AtCFL1* in the seedlings of (D) to (H). The expression level in wild types is set to 1.0. The error bars represent the SD of three biological replicates.

Because AtCFL1 interacted with HDG1 [58], CFLAP1 and CFLAP2, we would like to know which domain of AtCFL1 was responsible for these protein-protein interactions. We generated a series of truncated AtCFL1-GAL4 DNA binding domain constructs to test their interaction with GAL4 activation domain-fused CFLAP1 in YTH assay. As shown in Fig 9A, the full-length AtCFL1 protein and all of the truncated proteins including the C-terminal 50 amino acid residues could interact with CFLAP1, whereas that lacking the C-terminal 20 amino acid residues could not. This result suggested that the C-terminal 50 amino acid residues of AtCFL1 were required for AtCFL1–CFLAP1 interaction. Similar results were also obtained in the AtCFL1–HDG1 interaction (Fig 9A).

Within the sequence of the C-terminal 50 amino acid residues we found a putative C4 zinc finger domain (Fig 9B), which was reported to be important for protein–protein interaction [71, 72]. We made point mutations of the four cysteine residues, *i*.*e*., Cys^155^, Cys^158^, Cys^171^ and Cys^174^, into alanine and found that simultaneous loss of two adjacent zinc-interacting cysteine residues disrupted both the AtCFL1–CFLAP1 interaction and the AtCFL1–HDG1 interaction (Fig 9C), suggesting that these cysteine residues are necessary for these interactions. Similar results were also observed in the AtCFL1-CFLAP2 interaction (S8 Fig). Moreover, overexpression of cysteine-mutated AtCFL1, *i*.*e*., 35S:*AtCFL1C155AC158A*, 35S:*AtCFL1C171AC174A* and 35S:*C155AC1581C171AC174A* (35S:*AtCFL1C4m*), in wild-type *Arabidopsis* did not produce organ-fusion or TB staining phenotypes (Fig 9D to 9H), even though similar expression levels of 35S:*AtCFL1-myc-7* (S1 Fig) were observed in these transgenic plants (Fig 9I). These data suggested that the C-terminal C4 zinc finger domain was crucial for AtCFL1 to interact with different transcription factors and for its proper function in regulation of cuticle development in *Arabidopsis*.

**Fig 10:**
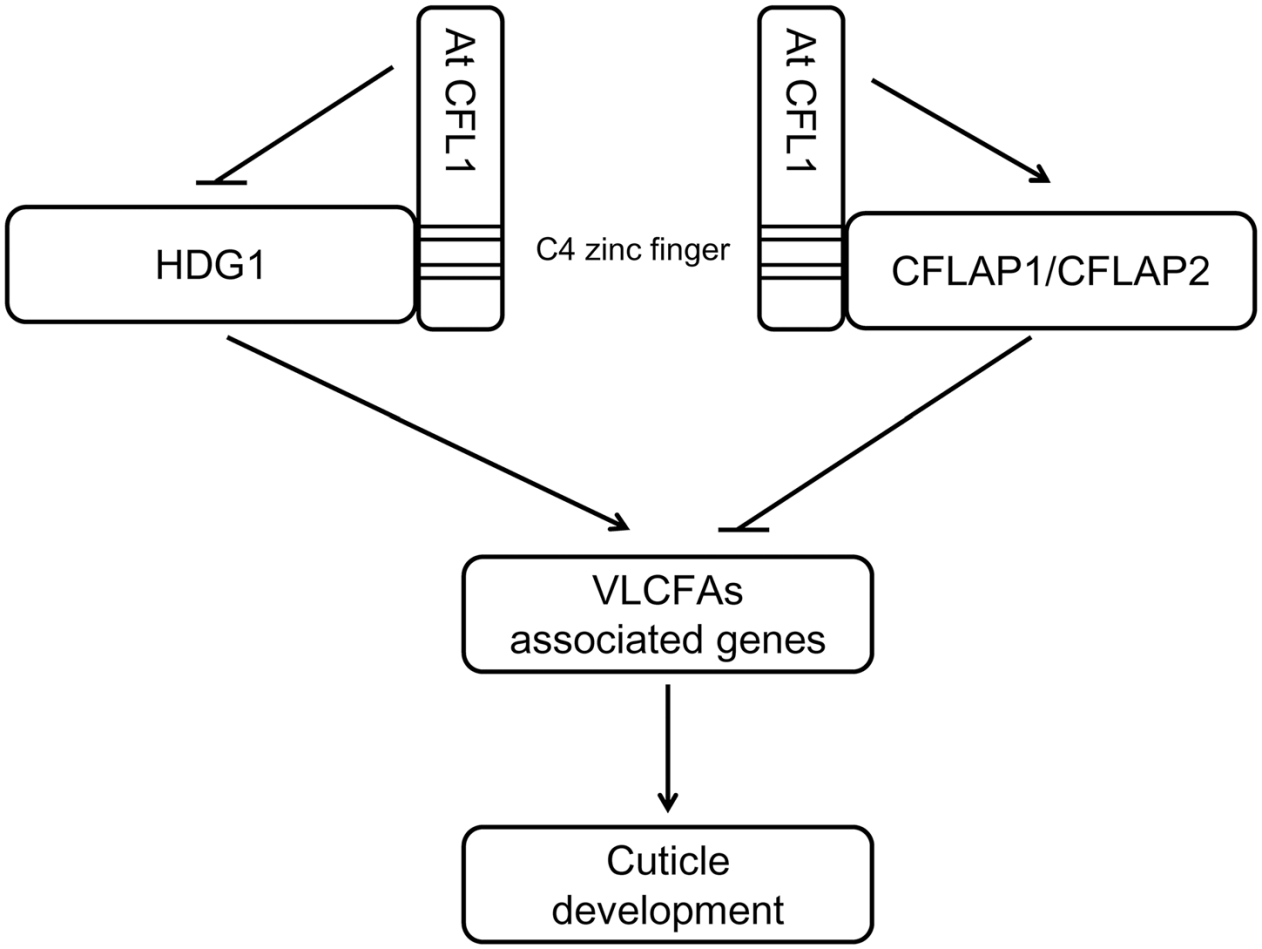
A working model of genetic relationships of AtCFL1 and other transcription factors. AtCFL1 regulates *Arabidopsis* cuticle development by interacting with transcription factors HDG1 and/or CFLAP1/2 via the same C-terminal zinc finger domain. HDG1 and CFLAP1/2 function in a synergistic but AtCFL1-dependent manner to maintain the balance of the expression of those genes involved in cuticle development.

In summary, as shown in the working model (Fig 10), AtCFL1 regulates *Arabidopsis* cuticle development by interacting with transcription factors HDG1 and/or CFLAP1/2 via the same C-terminal zinc finger domain. HDG1 and CFLAP1/2 function in a synergistic but AtCFL1-dependent manner to maintain the balance of the expression of those genes involved in cuticle development.

## Discussion

In this study, we have provided evidence to show that two bHLH transcription factors, CFLAP1 and CFLAP2, participate in synergistic regulation of AtCFL1-mediated cuticle development in *Arabidopsis*. First, both CFLAP1 and CFLAP2 interact with AtCFL1, a reported negative regulator of cuticle development in *Arabidopsis*, both *in vitro* and *in vivo*. Second, overexpression of either *CFLAP1* or *CFLAP2* causes cuticle defective phenotypes, including organ fusion, reduced epicuticular wax crystals in stems and wax accumulation in rosette leaves. Loss-of-function of *CFLAP1* and its homologs leads to opposite phenotypes. Third, the proper function of CFLAP1 is dependent on the presence of AtCFL1. Fourth, the C4 zinc finger domain of AtCFL1, the same domain responsible for interacting with HDG1, is also responsible for the interaction of AtCFL1 with CFLAP1/2. These results suggest that CFLAP1 and CFLAP2 represent two important transcription factors that are involved in synergistic regulation of AtCFL1-mediated cuticle development in *Arabidopsis*.

Different types of transcription factors have been reported to participate in the transcriptional regulation of cuticle development, *e*.*g*., AP2, MYB and HD-ZIP transcription factors [5, 45–55, 58, 73]. Now, a group of bHLH transcription factors can be added to this complex regulation network. Differing from those reported transcription factors that positively regulate cuticle development, these bHLH transcription factors are negative regulators, adding a new dimension to the regulatory network. Although, CFLAP1 and CFLAP2 are negative regulators of cuticle development in terms of genetics, this does not necessarily mean that they are direct repressors for cuticle development. Because CFLAP1 and CFLAP2 directly bind to the promoter region and activate the transcription of *CO* [60], it is plausible to hypothesize that the downstream target genes of CFLAP1 are probably negative regulators in cuticle development. We found from our RNA sequencing data that the transcriptional level of *DEWAX*, an AP2/ERF-type transcription factor gene recently identified as a repressor of wax biosynthesis [69], was increased in 35S:*CFLAP1* plants (Fig 6B). This suggests that *DEWAX* could be a candidate downstream target gene for CFLAP1, which will be verified in the future. The finding of opposite phenotypes in leaf and stem of both 35S:*CFLAP1* and 35S:*CFLAP1SRDX* plants was surprising, but not one of a kind: previously reported *lcr* and *fdh* mutants also exhibited different phenotypes in these two regions [18, 32, 64], suggesting that the molecular mechanism regulating the cuticle development might be not only complex but also organ-specific. Since *CFLAP1*, *CFLAP2* and *AtCFL1* are all expressed in vascular tissues, the possibility exists that they are involved in preventing cuticle formation within internal tissues of the plant, which needs to be investigated in the future.

It was reported that CFLAP1 and its homologs were phosphorylated under abscisic acid (ABA) treatment [70], suggesting a role for CFLAP1 in the ABA response. Phosphorylated CFLAP1 is released from the promoter of the down-stream gene *KAT1*, which encodes a K^+^ channel in guard cells. Previous reports showed that cuticular wax biosynthesis was activated under drought and ABA treatment [53]. Considering that CFLAP1 is a negative regulator of cuticle development, it is reasonable to predict that ABA, a signal in drought stress, possibly leads to phosphorylation of CFLAP1, which in turn disrupts the repressor genes and activates cuticle formation. Further investigation on whether the phosphorylation of CFLAP1 affects the CFLAP1–AtCFL1 interaction and/or the function of CFLAP1 in regulating cuticle development will help in elucidating how CFLAP1 works in the complex cuticle-regulating network.

Our study showed that the proper function of CFLAP1 was AtCFL1-dependent. AtCFL1 is a protein of 189 amino acid residues with a WW domain in the N-terminus and a C4 zinc finger domain in the C-terminus. Mutant analysis showed that the N-terminal WW domain is not important for the function of AtCFL1 in cuticle development. However, the C-terminal C4 zinc finger domain is responsible for AtCFL1–CFLAPs and AtCFL1–HDG1 interactions, and critical for AtCFL1 function, because overexpression of AtCFL1 with a mutated C4 zinc finger resulted in no obvious cuticle defective phenotypes. Zinc finger domains have been reported to be involved in transcriptional activation, DNA recognition, regulation of apoptosis and lipid binding [71, 72, 74, 75]. For instance, Ikaros, an essential regulator of lymphocyte differentiation, possesses a C-terminal zinc finger domain that is essential for its interaction with other proteins and for its DNA binding and transcriptional activation ability [71]. The fact that the C-terminal zinc finger domain of AtCFL1 interacted with both the negative regulators CFLAPs and the positive regulator HDG1 suggests that the regulation of cuticle development is probably temporal- and spatial-specific, or that the binding of different transcription factors to the zinc finger domain may be accomplished through binding competition. It also implies that AtCFL1 plays a central role in the transcriptional regulation network for cuticle development, by interacting with different types of transcription factors (*i*.*e*., HDG1 and CFLAPs). It will be interesting to clarify the biochemical nature of AtCFL1 and how it regulates the expression of downstream target genes during cuticle development.

## Methods

### Plant Materials and Growth Conditions

The *Arabidopsis thaliana* T-DNA insertion mutant SALK_074277 was described as *atcfl1-1* [58] and SALK_049022c was obtained from the ABRC. *Arabidopsis* plants ecotype Columbia-0 (Col-0) were used as wild type. The plants were grown on half-strength Murashige and Skoog (MS) medium containing 1% sucrose and 0.6% (w/v) phytoagar adjusted to pH5.7 using 1 M KOH, or in soil in greenhouse under long day condition with 16 hours light/ 8 hours dark cycle at 22±2°C. For the luciferase complementation assay and co-IP assay, *Nicotiana benthamiana* was grown in soil at 22 ± 2°C under long day condition.

### Vector Construction and Transformation

The coding region of *AtCFL1* was cloned into pENTR/D-TOPO (Invitrogen) using the primers AtCFL1-TOPO-F and AtCFL1-TOPO-R to generate pENTR-AtCFL1. pENTR-AtCFL1 was cloned into pDEST32 (Invitrogen) by LR reaction, generating plasmid pDEST32-AtCFL1 as bait in yeast two-hybrid assay. The coding region of CFLAP1 was cloned into pENTR/D-TOPO using the primers CFLAP1-TOPO-F and CFLAP1-TOPO-R to form pENTR-CFLAP1. The prey construct of pDEST22-CFLAP1 was generated by LR reaction between pENTR-CFLAP1 and pDEST22 (Invitrogen). For the constructs for firefly luciferase complementation imaging assay, the *Kpn* I-*Sal* I fragment of *AtCFL1* was ligated into *Kpn* I-*Sal* I fragment of plasmid pCAMBIA-CLuc [76] to generate AtCFL1-cLUC plasmid. The *Bam*H I-*Sal* I fragment of *CFLAP1* and *Bam*H I-*Sal* I fragment form pCAMBIA-NLuc [76] were ligated to form plasmid CFLAP1-nLUC. For the co-IP experiment, construct of *AtCFL1-myc* was generated from LR reaction between pENTR-AtCFL1 without stop codon and pK7MYCGW2 (from Li-Jia Qu lab). The plasmid GFP-CFLAP1 was generated from LR reaction between pENTR-CFLAP1 and pK7WGF2.

For the plasmids with truncated AtCFL1 proteins used in yeast two-hybrid assay, the different fragments of *AtCFL1* were cloned into pENTR/D-TOPO at first, using the primers combinations as follow: AtCFL1 TOPO-F/ AtCFL1 N70 TOPO-R, AtCFL1 C119 TOPO-F/ AtCFL1 TOPO-R, AtCFL1 C98 TOPO-F/ AtCFL1 TOPO-R, AtCFL1 C77 TOPO-F/ AtCFL1 TOPO-R, AtCFL1 C50 TOPO-F/ AtCFL1 TOPO-R and AtCFL1 TOPO-F/ AtCFL1 ΔC20 TOPO-R. These pENTR plasmids were cloned into pDEST32 by LR reactions. The point mutants of AtCFL1 were made by using the Easy Mutagenesis System (TransGen Biotech).

*CFLAP1* overexpression construct was generated by LR reaction between pENRT-CFLAP1 and pB2GW7. For the chimeric repressor construct, the *CFLAP1* coding sequence was amplified from *Arabidopsis* cDNA by RT-PCR used the primers CFLAP1 SRDX-F and CFLAP1 SRDX-R. This *Bgl* II-*Spe* I fragment was ligated into pC35SSRDX *Bgl* II-*Spe* I plasmid to generate CFLAP1-SRDX construct.

Constructs were transformed into *Agrobacterium tumefaciens* GV3101 using the freeze-thaw procedure and then into *Arabidopsis* as described previously [77].

All primers used in this study are listed in S3 Table.

### Yeast Two-Hybrid Assay

The bait plasmid pDEST32-AtCFL1 was transformed into yeast train AH109. The resulting yeast was used for a mating-based yeast two-hybrid screening for a transcription factors library of *Arabidopsis* as described [59].

For the interaction of AtCFL1 and CFLAP1 homologs, the coding regions of *CFLAP2/FBH1*, *FBH2* and *FBH4* were amplified from *Arabidopsis* cDNA by RT-PCR, and cloned into pDEST22 as preys respectively. Bait plasmid pDEST32-AtCFL1 and preys or the blank pDEST22 were co-transformed into yeast strain AH109, respectively.

Medium supplemented with SD-Leu-Trp-His and SD-Leu-Trp-His add 5 mM 3-amino-1,2,4 triazole were used for selection. Three biological replicates of all experiments were conducted.

### Firefly Luciferase Complementation Imaging Assay and Co-IP Assay

The firefly luciferase complementation imaging assay was conducted according to the protocol by Chen et al. [76]. The different plasmid combinations, *e*.*g*., AtCFL1-cLUC and CFLAP1-nLUC, AtCFL1-cLUC and nLUC, cLUC and CFLAP1-nLUC, cLUC and nLUC, were co-infiltrated into tobacco (*Nicotiana benthamiana*) leaves as described by Wu et al. [58]. The tobacco plants were incubated in dark for 24 hours and then put back in green house for 24–48 hours in normal long-day light condition. The leaves were cut and sprayed with luciferin (100 mM) and then kept in the dark for 15 min, then observed under a low-light cooled charge-coupled device (CCD) imaging apparatus Lumazone 1300B (Roper Bioscience).

For the co-IP experiment, the constructs *AtCFL1-myc* and *GFP-CFLAP1* were infiltrated into tobacco leaves. The following experiment was performed as described previously [58]. Two biological replicates of the experiments were conducted.

### Microscopy

For the pollen germination assay, wild-type pollen was applied to abaxial and adaxial surfaces of 4-week-old rosette leaves of wild type and 35S:*CFLAP1* plants respectively. The plants were grown in green house for 24 hours and the leaves were removed and fixed in the FAA buffer containing 50% ethanol, 6% glacial acetic acid, and 5% formaldehyde for 4 hours at room temperature. Serial ethanol dehydration was performed. Then samples were dried at critical point in liquid CO_2_ and coated with gold powder. The inflorescence stems were dried in incubator and mounted directly. These samples were analyzed using a scanning electron microscope (JEOL JSM-6610LV) as described in the user manual. For the Cryo-SEM, samples were glued on a sample holder of cryo-transfer system (ALTO 1000, Gatan UK) and frozen in liquid nitrogen. The samples were transferred under vacuum and incubated in cryo-preparation chamber, stay in -90°C for 5 min. Then the sample surface was sputter coated and observed under cold stage (-150°C).

### Total RNA Isolation and Quantitative RT-PCR Analysis

Total RNA was extracted from frozen material using TRIzol reagent (Invitrogen) and then treated with RNase-free DNase (TaKaRa) to remove the genomic DNA. Five micrograms of total RNA was reversely transcribed using the M-MLV kit (Invitrogen) for each sample. The cDNA was diluted and used as template for RT-PCR or quantitative RT-PCR. Quantitative RT-PCR was performed using SYBR Green real-time PCR Master Mix (Toyobo) as described previously [78]. The relative expression level of each gene was calculated using the 2 –^ΔΔCt^ (cycle threshold) method [79], and *TUB2* was used as an internal control. At least three repeats were performed for each sample.

### Toluidine Blue Staining Test and Quantificational Analysis

The 14-day-old seedlings or the 3-week-old rosette leaves of *Arabidopsis* were used for the TB staining as described [61]. The plant materials were immersed in 0.05% solution of TB at room temperature for 2 minutes, and then washed with water for three times.

The quantificational analysis of TB staining was performed as described by Tanaka et al. [80] with slight modification. The aerial parts of 14-day-old seedlings were cut after TB staining, and ground in 200 μL buffer [200 mM Tris-HCl (pH8.0), 250 mM NaCl, 25 mM EDTA] and 400 μL ethanol was added. After vortex mixing and centrifugation, the supernatant was examined by spectrophotometer for the absorbance at 630 nm (A_630_) and 435 nm (A_435_). Relative levels of absorbed TB were calculated as the ratio of A_630_:A_435_.

### Epicuticular Wax Analysis

The analysis was performed as described by Xia et al. [81] with some modification. *Arabidopsis* stems or rosette leaves were immersed in 10 mL chloroform, adding 50 μL *n*-tetracosane (20 μg/mL) as an internal standard, for 30 seconds at 60°C to extract epicuticular wax. Five stems and seven rosette leaves were used for each replicate. The extract solution was evaporated under a stream of nitrogen. By adding 30 μL pyridine and 50 μL N,O-bis (trimethylsilyl) fluoroacetamide (BSTFA), samples were incubated at 70°C for 1 hour. After derivatization, samples were dissolved in 100 μL *n*-hexane for GC-MS analysis. The 6-week-old 35S:*CFLAP1* plants, 12-week-old 35S:*CFLAP1-SRDX* plants and the contemporaneous wild-type plants were used for the waxes extraction, respectively.

### RNA Sequencing Analysis

Total RNA was extracted from the aerial parts of 3-week-old wide-type and 35S:CFLAP1 plants using TRIzol reagent (Invitrogen). RNA sequencing was performed by Illumina HiSeq 2000 at the Biodynamic Optical Imaging Center (BIOPIC) in Peking University. The resulting data was analyzed according the procedures described previously [82]. In briefly, RNA-seq reads generated by Illumina Hiseq 2000 for each library were mapped independently using TopHat version 2.0.6 (http://ccb.jhu.edu/software/tophat/index.shtml) against *Arabidopsis thaliana* genome sequence index (Ensembl, TAIR 10 version), downloaded from Illumina iGenome (http://ccb.jhu.edu/software/tophat/igenomes.shtml). For differential gene analyses of two samples, Cuffdiff version 2.0.1 was run by using the reference transcriptome along with the BAM files resulting from Tophat for each sample. The gene_exp.diff file generated by cuffdiff program lists the results of differential expression testing between the two samples at gene level. For the selection of up-regulated genes in the transgenic plants, the criteria was set: FPKM _WT_> = 0.2, log2 (FPKM _35:CFLAP1 (or 35:CFLAP1SRDX)_ /FPKM _WT_) < = -1; while for selecting down-regulated genes, the criteria was set: FPKM_35:CFLAP1 (or 35:CFLAP1SRDX)_ > = 0.2, log2 (FPKM _35:CFLAP1 (or 35:CFLAP1SRDX)_ /FPKM _WT_) > = 1.

### Accession Numbers

Sequence data referred in this study can be found in the *Arabidopsis* Genome Initiative, GenBank/EMBL/UniProtKB databases, or Rice Genome Annotation Project under the following accession numbers: *AtCFL1*, *At2g33510*; *CFLAP1*, *At1g51140*; *CFLAP2*, *At1g35460*; *HDG1*, *At3g61150*; *FBH2*, *At4g09180*; *FBH4*, *At2g42280*; *FDH*, *At2g26250; BDG*, *At1g64670; KCS8*, *At2g15090; DEWAX*, *At5g61590; TUB2*, *At5g62690; M4CMQ1*; *B9GZB4*; *K7MVM7*; *D7U611*; *B6TJV0*; *B7FMU4*; *CFL1*, *Os02g31140*.

## Supporting Information

S1 Fig The fusion proteins of AtCFL1-myc and GFP-CFLAP1 are functional. (A) TB staining assay of 14-day-old seedlings. Left, before TB staining; right, after TB staining for 2 minutes. From top to bottom, wild type, 35S:*AtCFL1-myc-1*, 35S:*AtCFL1-myc-7*, respectively. Bar = 1 mm. (B) TB staining assay of rosette leaf. Left, before TB staining; right, after TB staining for 2 minutes. From top to bottom, wild type, 35S:*GFP-CFLAP1-1*, 35S: *GFP-CFLAP1-2*, respectively. Bar = 1 mm. (C) and (D) SEM images of the epicuticular wax crystals on the stems of wild type and 35S:*GFP-CFLAP1-1*. Bar = 5 μm.
(TIF)

S2 Fig Other phenotypes of 35S:*CFLAP1* plants. (A) Early-flowering phenotype of the 35S:*CFLAP1* plants. Left, wild type; right, 35S:*CFLAP1* plants. (B) Abnormal flowers in the 35S:*CFLAP1* plants. Bar = 1 mm. (C) Abnormal siliques in the 35S:*CFLAP1* plants. Bar = 1 mm. (D) Leaf numbers of flowering for wild type, 35S:*CFLAP1-1* and 35S:*CFLAP1-3* plants. Level of significance obtained with a Student’s *t* test indicated by ***, p<0.01. (E) Relative expression level of *CFLAP1* in wild type, 35S:*CFLAP1-1* and 35S:*CFLAP1-3* plants. The expression level in wild types is set to 1.0. The error bars represent the SD of three biological replicates. (F) and (G) TEM images of rosette leaves of wild-type and 35S: *CFLAP1* respectively. Arrows indicate the cutin layer. Bar = 200 nm.
(TIF)

S3 Fig The overexpression of *FBH2* and *FBH4* did not cause cuticle defective phenotype. (A) The result of yeast two-hybrid assay for the interactions of FBH2-AtCFL1 and FBH4-AtCFL1. The co-transformed yeast was plated on the control medium SD-LW and selective medium SD-LWH plus 3-amino-1, 2, 4-triazole (3-AT). HDG1 was used as a positive control. (B) to (F) TB staining assay of 14-day-old seedlings. (B), wild type; (C), 35S:*CFLAP2-27*; (D), 35S:*FBH2-3*; (E), 35S:*CFLAP1-3*; (F), 35S:*FBH4-7*. The plants of 35S:*CFLAP2-27* and 35S:*CFLAP1-3* could be stained to blue, while the plants of 35S:*FBH2-3* and 35S:*FBH4-7* could not. Bar = 1mm.
(TIF)

S4 Fig The phenotypes of the *fbh1 fbh2 fbh3 fbh4* quatruple mutant. (A) The phylogenetic tree of *CFLAP1* and its three homologous genes. (B) Relative expression levels of *CFLAP1/FBH3*, *CFLAP2/FBH1*, *FBH2* and *FBH4* in the wild type and quadruple mutant. The expression level in the wild type is set to 1.0, and error bars represent the SD of three biological replicates. (C) Epicuticular wax components in stems of quadruple mutant and wild type. Numbers indicate the main chain lengths of each constituent. Each value is the mean + SD of five biological replicates. At least 4 independent stems were used for each replicate. (D) Epicuticular wax components in rosette leaves of the *fbh1 fbh2 fbh3 fbh4* quadruple mutant and wild type. Numbers indicate the main chain lengths of each constituent. Each value is the mean + SD of five biological replicates. At least 5 rosette leaves from different independent plants were used for each replicate. Level of significance obtained with a Student’s *t* test is marked by the following: *, p<0.05.
(JPG)

S5 Fig Other phenotypes of 35S:*CFLAP1SRDX* plants. (A) Late-flowering phenotype of 35S:*CFLAP1SRDX-48* plants. Left, 35S:*CFLAP1SRDX-48* plants; right, wild type. (B) Leaf numbers for the flowering plants of 35S:*CFLAP1SRDX-48* and wild type. (C) and (D) SEM images of the epicuticular wax crystals on inflorescence stems of 35S:*CFLAP1SRDX-48* and wild type. Bar = 5 μm.
(TIF)

S6 Fig The change of epicuticular waxes showed similar trends in 35S:*CFLAP1SRDX-41* and 35S: *CFLAP1SRDX-48* plants. (A) Epicuticular wax components of 35S: *CFLAP1SRDX-41*, 35S: *CFLAP1SRDX-48* and wild-type stems. Numbers indicate the main chain length of each constituent. Each value is the mean + SD of three biological replicates. At least 4 independent stems were used for each replicate. (B) Epicuticular wax components of 35S: *CFLAP1SRDX-41*, 35S: *CFLAP1SRDX-48* and wild-type rosette leaves. Numbers indicate the main chain length of each constituent. Each value is the mean + SD of three biological replicates. At least 5 rosette leaves from different independent plants were used for each replicate. Level of significance obtained with a Student’s *t* test is marked by the following: *, p<0.05; ***, p<0.01.
(TIF)

S7 Fig The subcellular localization of CFLAP1. (A) GFP signal of 35S:*GFP-CFLAP1* plant root tip. (B) DAPI stained root tip. (C) Bright field. (D) (A) to (C) merged together. Bars = 30 μm.
(TIF)

S8 Fig The putative zinc finger domain in the AtCFL1 C-terminus is necessary for AtCFL1–CFLAP2 interaction. The results of yeast two-hybrid for the interactions between CFLAP2 and mutated AtCFL1s. The baits were wild-type AtCFL1, AtCFL1 with C155 and C158 residues mutated, AtCFL1 with C171 and C174 residues mutated and AtCFL1 with C155, C158, C171 and C174 residues mutated respectively. The co-transformed yeast strains were plated on the control medium SD-LW and selective medium SD-LWH.
(TIF)

S1 Table The result of 35:*CFLAP1* RNA-seq data. (XLS)

S2 Table The result of 35:*CFLAP1SRDX* RNA-seq data. (XLS)

S3 Table Primer information used in this study. (DOC)
