# Supplementary figures and images for: CFLAP1 and CFLAP2 Are Two bHLH Transcription Factors Participating in Synergistic Regulation of AtCFL1-Mediated Cuticle Development in Arabidopsis
Source: PLoS Genet. 2016 Jan 8;12(1):e1005744. doi: 10.1371/journal.pgen.1005744 (PMC4706423; doi:10.1371/journal.pgen.1005744)

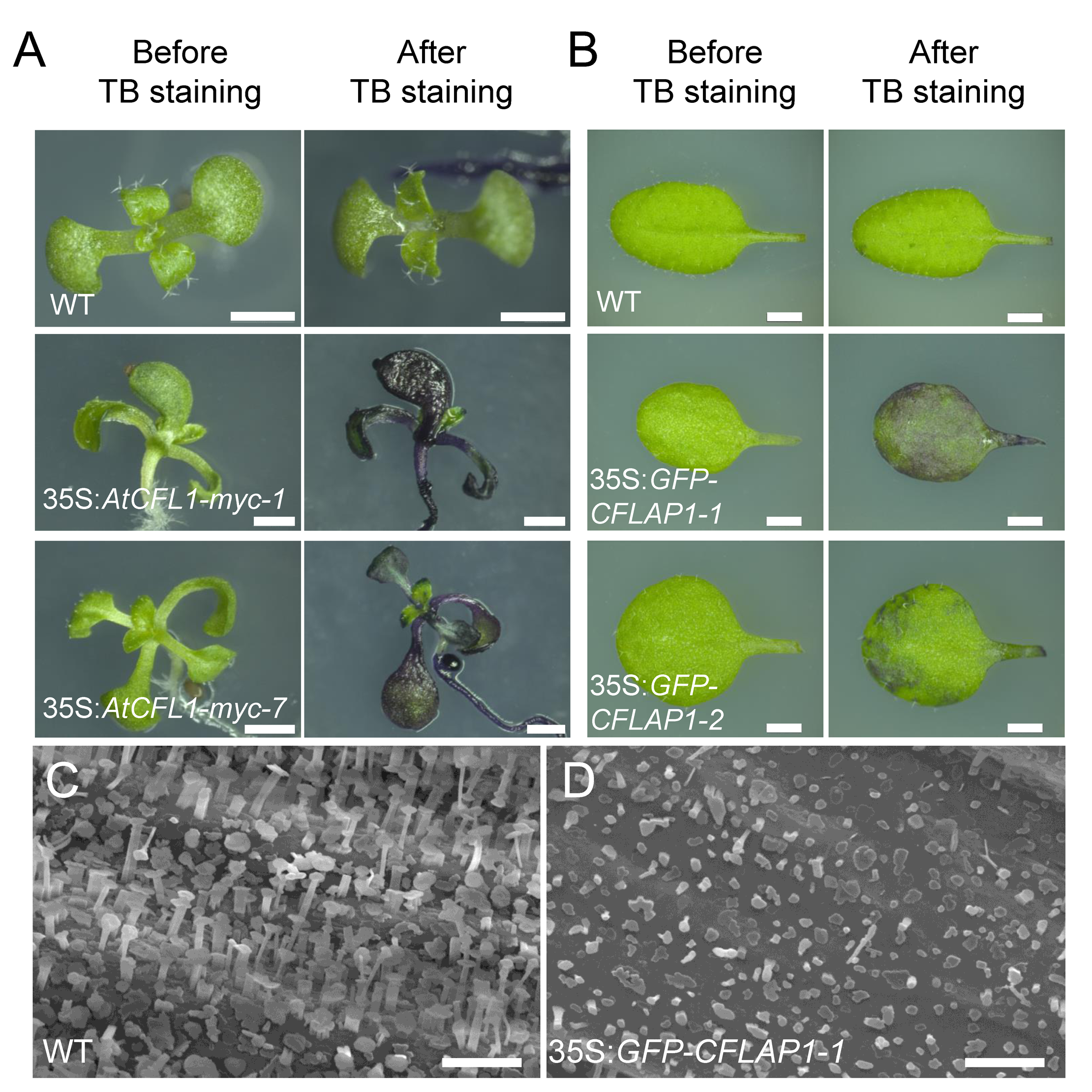

Supplement: S1 Fig — (A) TB staining assay of 14-day-old seedlings. Left, before TB staining; right, after TB staining for 2 minutes. From top to bottom, wild type, 35S:AtCFL1-myc-1, 35S:AtCFL1-myc-7, respectively. Bar = 1 mm. (B) TB staining assay of rosette leaf. Left, before TB staining; right, after TB staining for 2 minutes. From top to bottom, wild type, 35S:GFP-CFLAP1-1, 35S: GFP-CFLAP1-2, respectively. Bar = 1 mm. (C) and (D) SEM images of the epicuticular wax crystals on the stems of wild type and 35S:GFP-CFLAP1-1. Bar = 5 μm. (TIF) [file pgen.1005744.s001.tif]

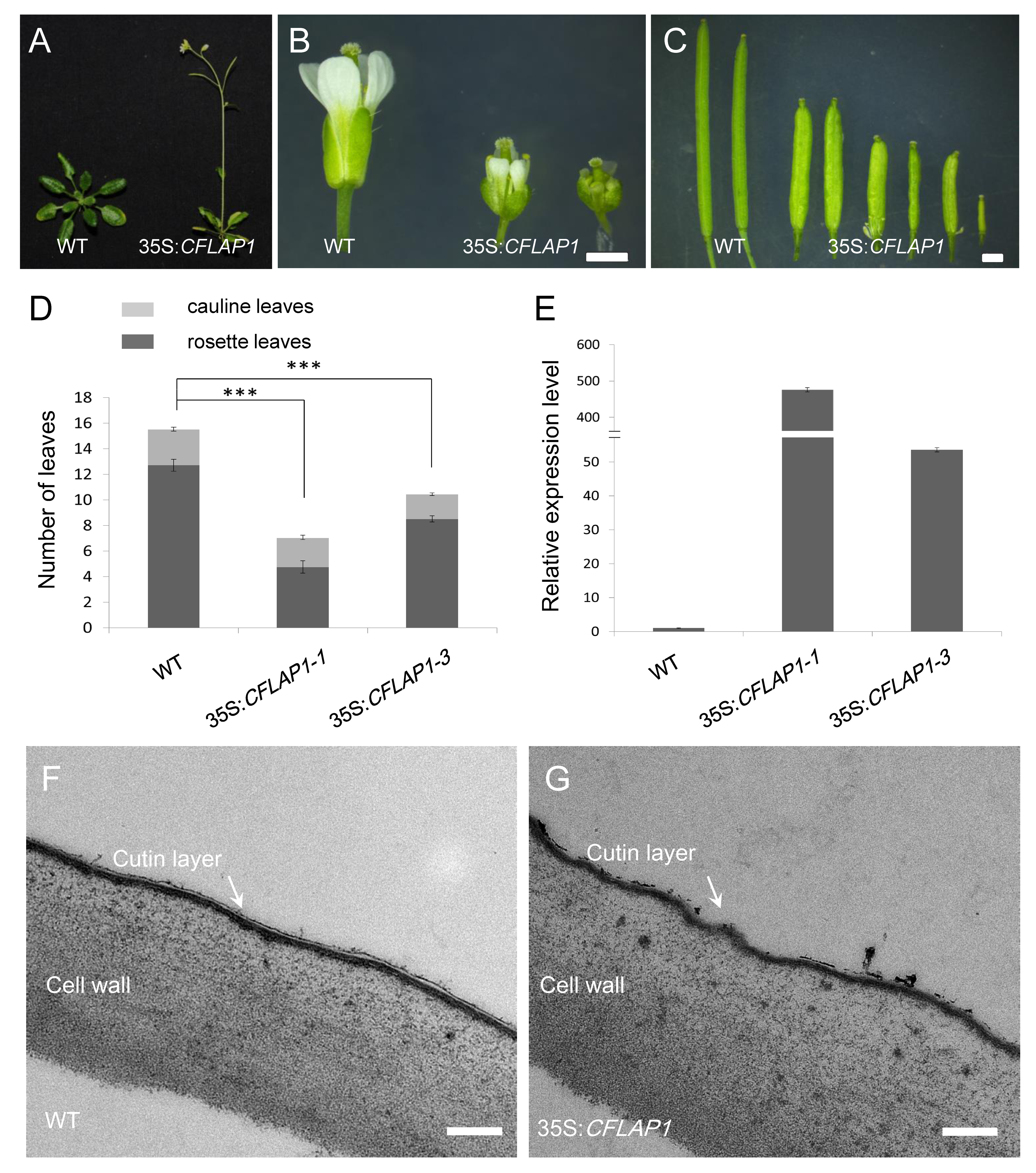

Supplement: S2 Fig — (A) Early-flowering phenotype of the 35S:CFLAP1 plants. Left, wild type; right, 35S:CFLAP1 plants. (B) Abnormal flowers in the 35S:CFLAP1 plants. Bar = 1 mm. (C) Abnormal siliques in the 35S:CFLAP1 plants. Bar = 1 mm. (D) Leaf numbers of flowering for wild type, 35S:CFLAP1-1 and 35S:CFLAP1-3 plants. Level of significance obtained with a Student’s t test indicated by ***, p<0.01. (E) Relative expression level of CFLAP1 in wild type, 35S:CFLAP1-1 and 35S:CFLAP1-3 plants. The expression level in wild types is set to 1.0. The error bars represent the SD of three biological replicates. (F) and (G) TEM images of rosette leaves of wild-type and 35S: CFLAP1 respectively. Arrows indicate the cutin layer. Bar = 200 nm. (TIF) [file pgen.1005744.s002.tif]

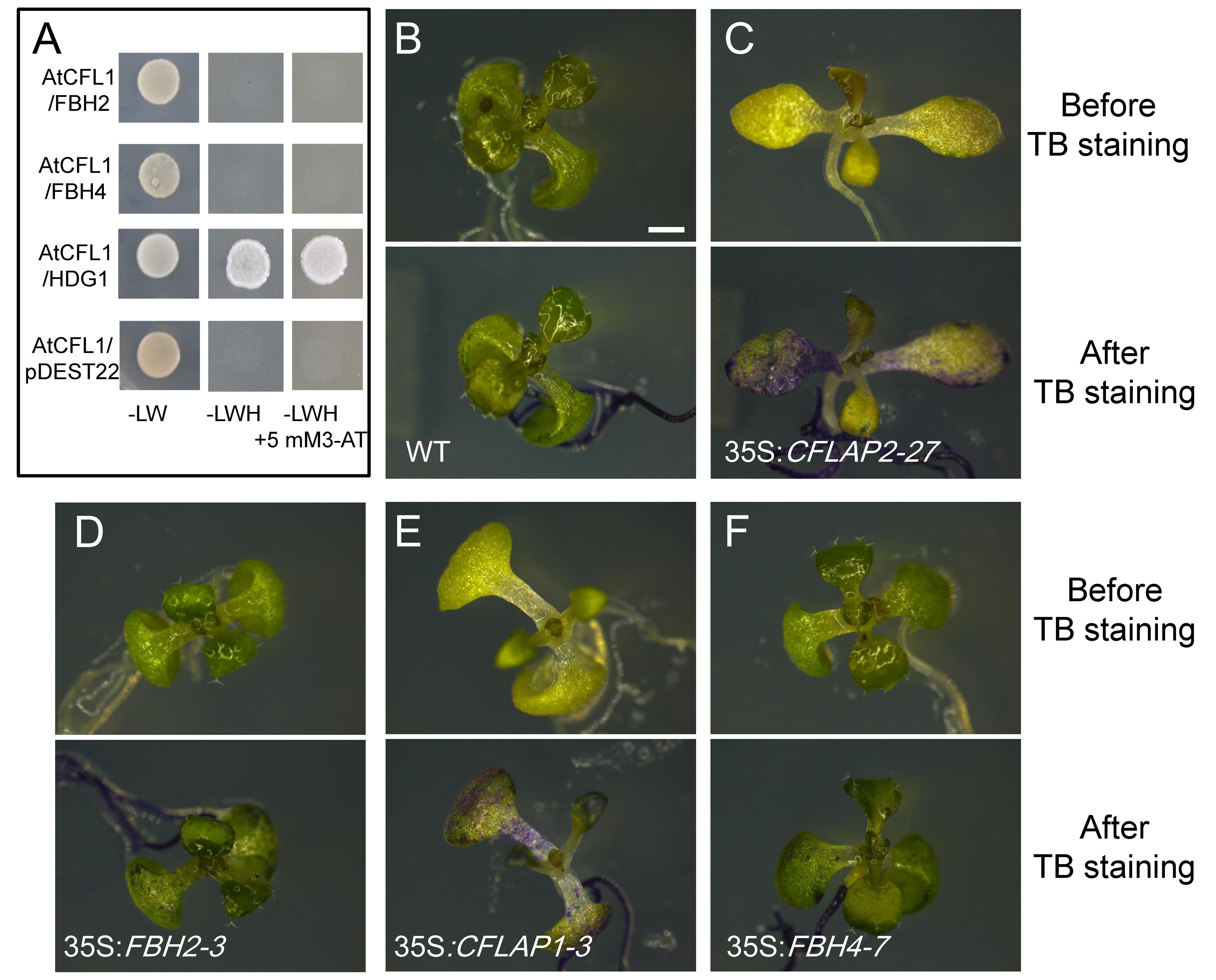

Supplement: S3 Fig — (A) The result of yeast two-hybrid assay for the interactions of FBH2-AtCFL1 and FBH4-AtCFL1. The co-transformed yeast was plated on the control medium SD-LW and selective medium SD-LWH plus 3-amino-1, 2, 4-triazole (3-AT). HDG1 was used as a positive control. (B) to (F) TB staining assay of 14-day-old seedlings. (B), wild type; (C), 35S:CFLAP2-27; (D), 35S:FBH2-3; (E), 35S:CFLAP1-3; (F), 35S:FBH4-7. The plants of 35S:CFLAP2-27 and 35S:CFLAP1-3 could be stained to blue, while the plants of 35S:FBH2-3 and 35S:FBH4-7 could not. Bar = 1mm. (TIF) [file pgen.1005744.s003.tif]

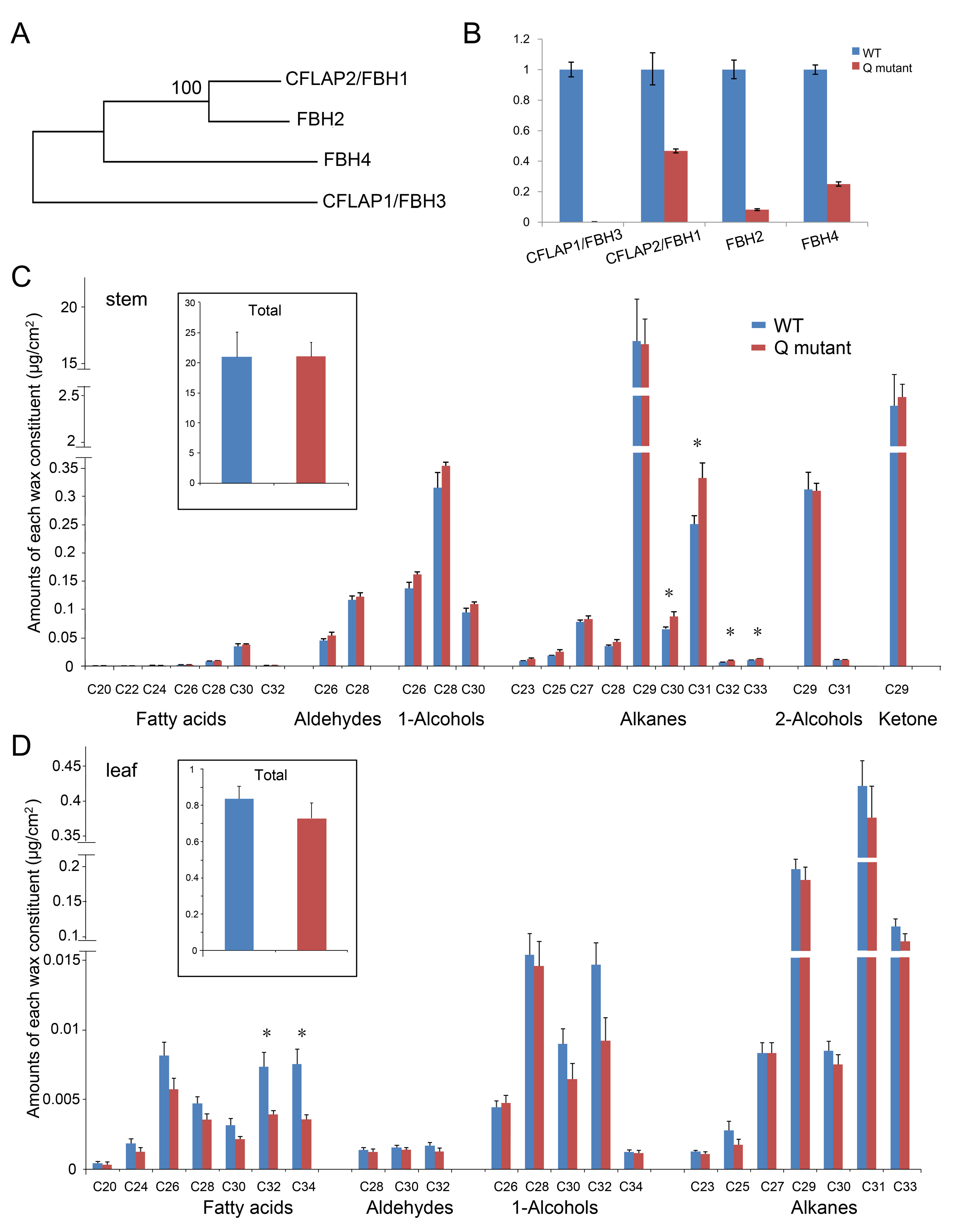

Supplement: S4 Fig — (A) The phylogenetic tree of CFLAP1 and its three homologous genes. (B) Relative expression levels of CFLAP1/FBH3, CFLAP2/FBH1, FBH2 and FBH4 in the wild type and quadruple mutant. The expression level in the wild type is set to 1.0, and error bars represent the SD of three biological replicates. (C) Epicuticular wax components in stems of quadruple mutant and wild type. Numbers indicate the main chain lengths of each constituent. Each value is the mean + SD of five biological replicates. At least 4 independent stems were used for each replicate. (D) Epicuticular wax components in rosette leaves of the fbh1 fbh2 fbh3 fbh4 quadruple mutant and wild type. Numbers indicate the main chain lengths of each constituent. Each value is the mean + SD of five biological replicates. At least 5 rosette leaves from different independent plants were used for each replicate. Level of significance obtained with a Student’s t test is marked by the following: *, p<0.05. (JPG) [file pgen.1005744.s004.jpg]

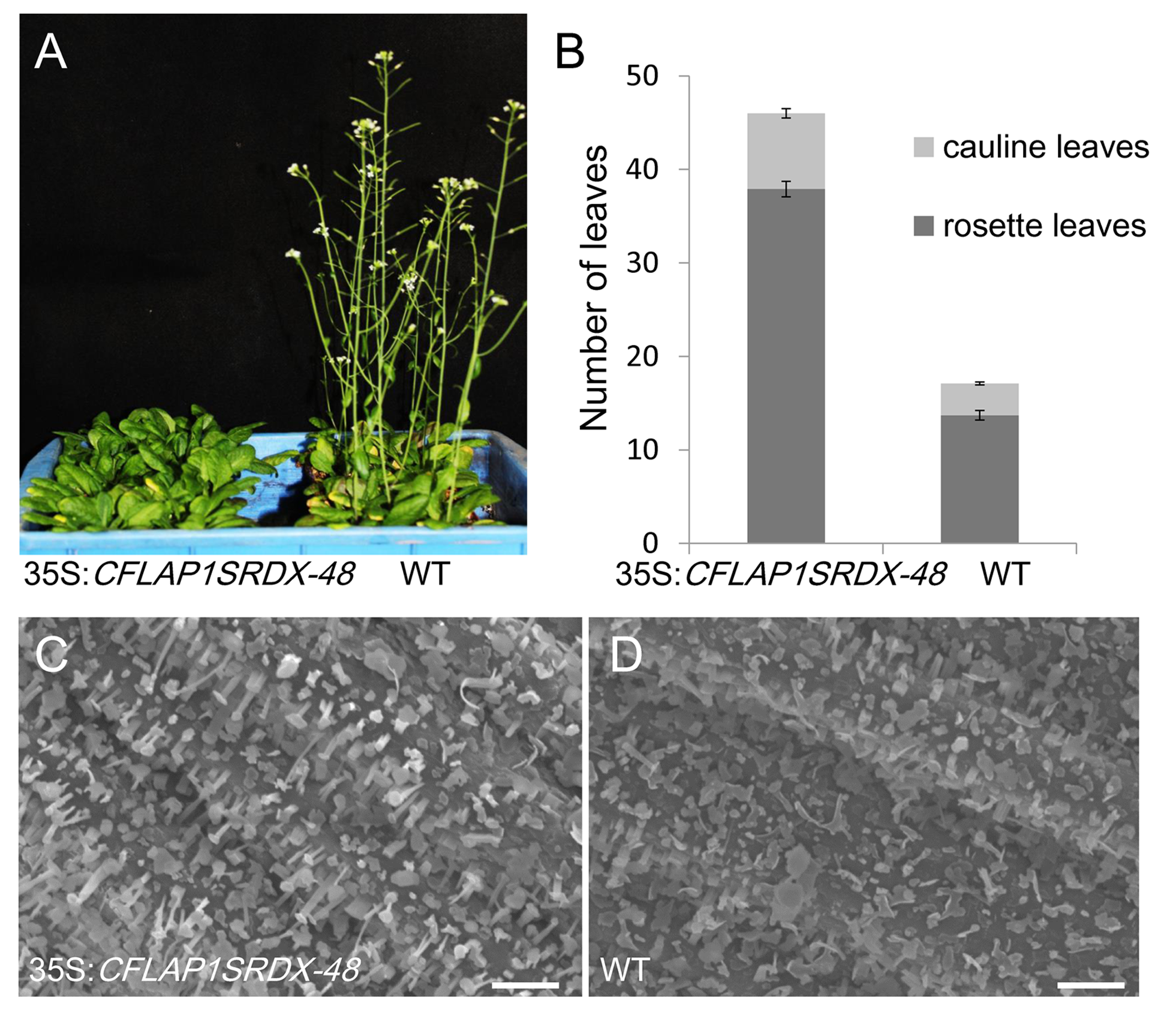

Supplement: S5 Fig — (A) Late-flowering phenotype of 35S:CFLAP1SRDX-48 plants. Left, 35S:CFLAP1SRDX-48 plants; right, wild type. (B) Leaf numbers for the flowering plants of 35S:CFLAP1SRDX-48 and wild type. (C) and (D) SEM images of the epicuticular wax crystals on inflorescence stems of 35S:CFLAP1SRDX-48 and wild type. Bar = 5 μm. (TIF) [file pgen.1005744.s005.tif]

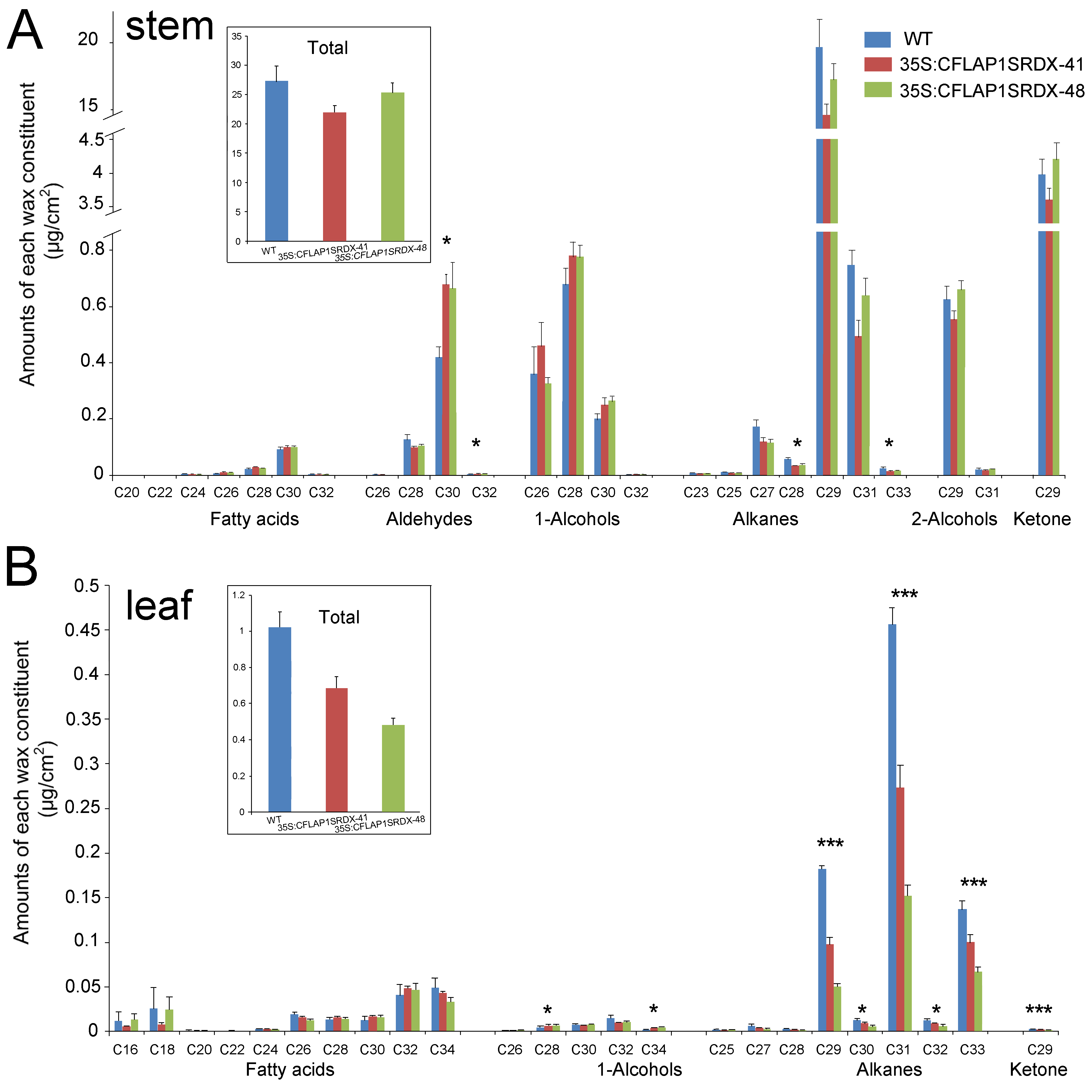

Supplement: S6 Fig — (A) Epicuticular wax components of 35S: CFLAP1SRDX-41, 35S: CFLAP1SRDX-48 and wild-type stems. Numbers indicate the main chain length of each constituent. Each value is the mean + SD of three biological replicates. At least 4 independent stems were used for each replicate. (B) Epicuticular wax components of 35S: CFLAP1SRDX-41, 35S: CFLAP1SRDX-48 and wild-type rosette leaves. Numbers indicate the main chain length of each constituent. Each value is the mean + SD of three biological replicates. At least 5 rosette leaves from different independent plants were used for each replicate. Level of significance obtained with a Student’s t test is marked by the following: *, p<0.05; ***, p<0.01. (TIF) [file pgen.1005744.s006.tif]

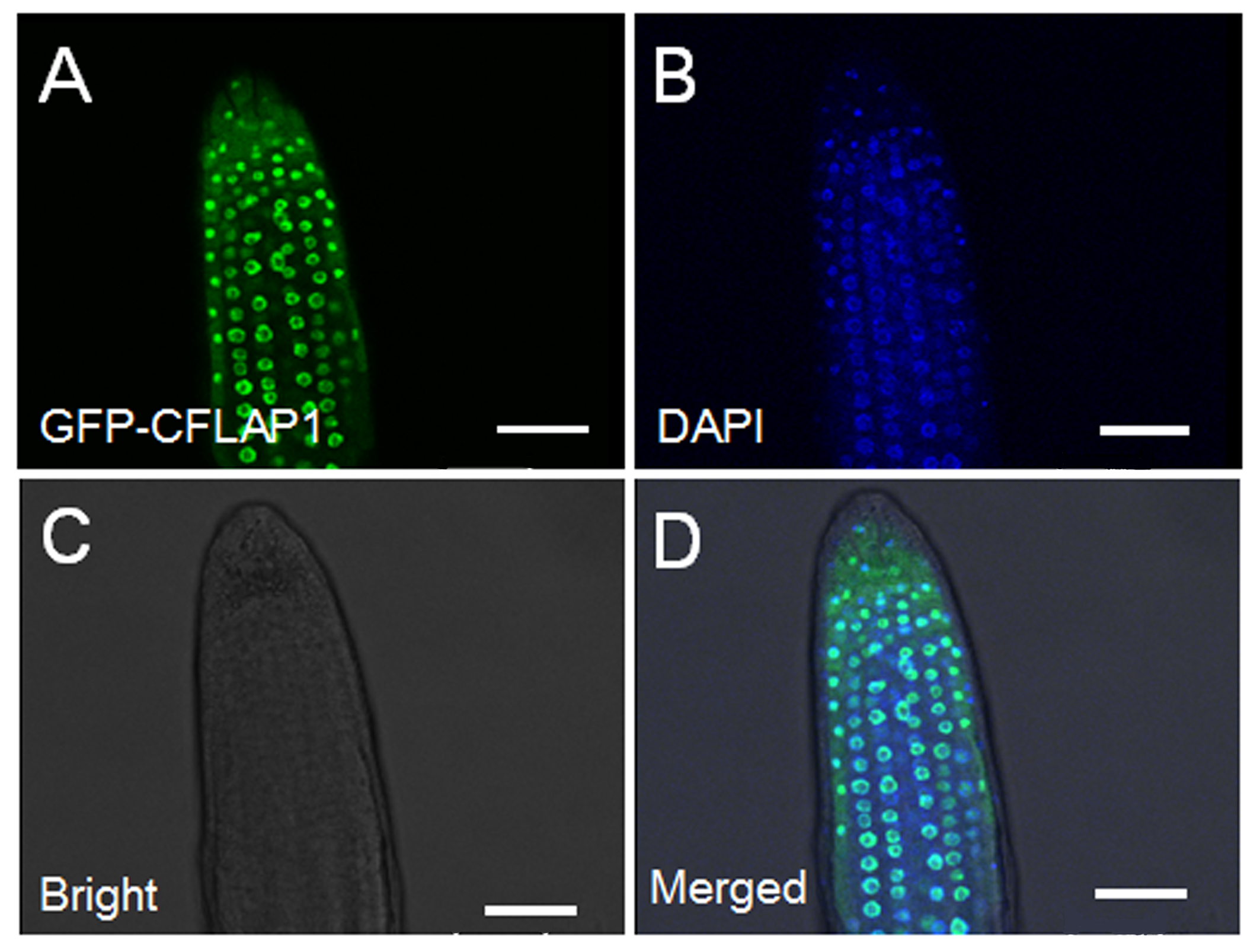

Supplement: S7 Fig — (A) GFP signal of 35S:GFP-CFLAP1 plant root tip. (B) DAPI stained root tip. (C) Bright field. (D) (A) to (C) merged together. Bars = 30 μm. (TIF) [file pgen.1005744.s007.tif]

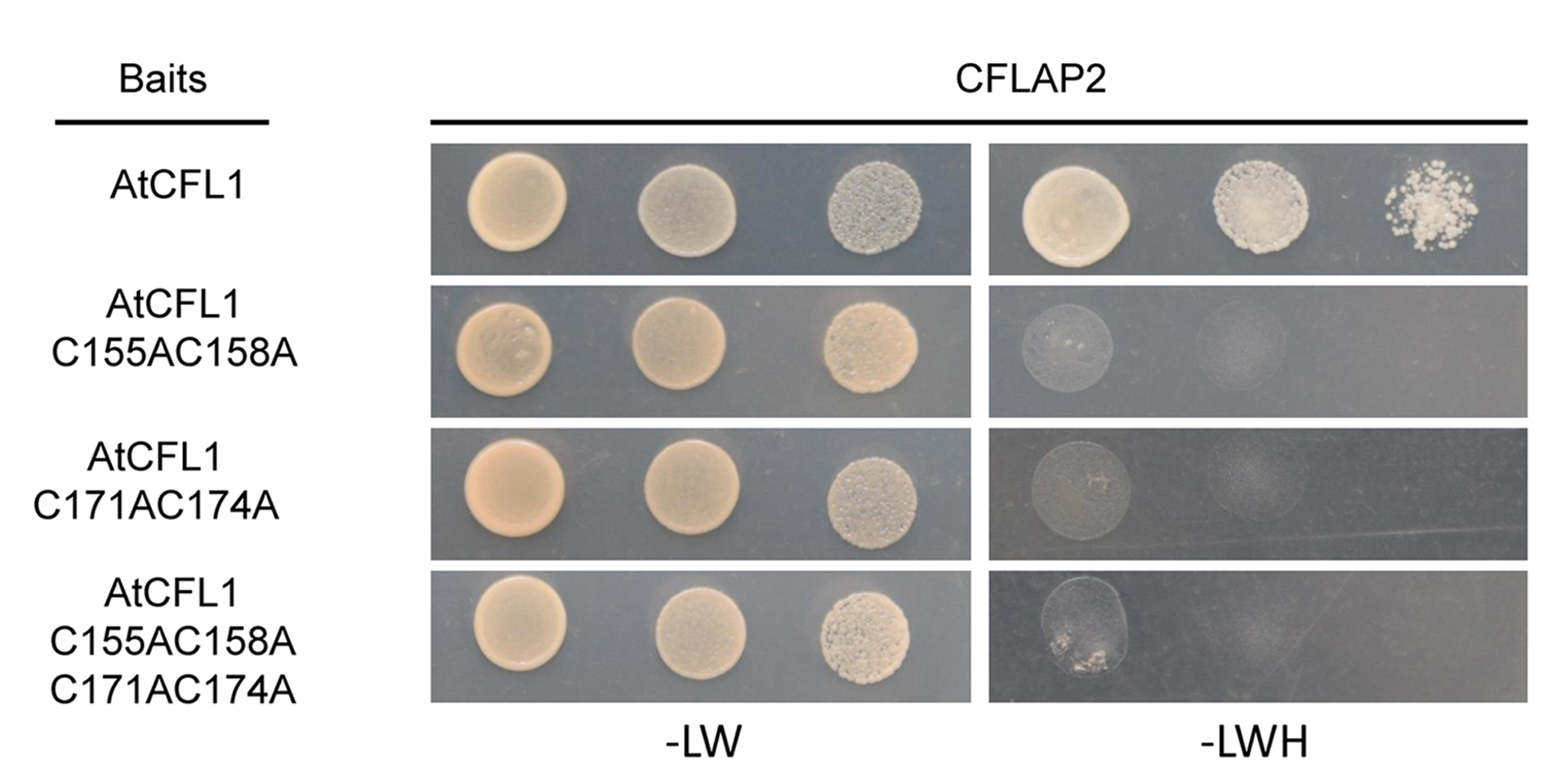

Supplement: S8 Fig — The results of yeast two-hybrid for the interactions between CFLAP2 and mutated AtCFL1s. The baits were wild-type AtCFL1, AtCFL1 with C155 and C158 residues mutated, AtCFL1 with C171 and C174 residues mutated and AtCFL1 with C155, C158, C171 and C174 residues mutated respectively. The co-transformed yeast strains were plated on the control medium SD-LW and selective medium SD-LWH. (TIF) [file pgen.1005744.s008.tif]
