# Supplementary material for: CFLAP1 and CFLAP2 Are Two bHLH Transcription Factors Participating in Synergistic Regulation of AtCFL1-Mediated Cuticle Development in Arabidopsis
Source: PLoS Genet. 2016 Jan 8;12(1):e1005744. doi: 10.1371/journal.pgen.1005744 (PMC4706423; doi:10.1371/journal.pgen.1005744)
Supplement: S3 Table — (DOC) [file pgen.1005744.s011.doc]

| Table S3. Primer information used in this study | |
| --- | --- |
| Name | Sequence 5’-------------------------- 3’ |
| AtCFL1-TOPO-F | CACCATGAAAGCACCCAACA |
| AtCFL1-TOPO-R | TCAAGAAGATGCAGAATGAG |
| CFLAP1-TOPO-F | CACCATGGAATCAGAATTCCAGCAACATC |
| CFLAP1-TOPO-R | TCACGCACTAGAGCATCTACATCTT |
| AtCFL1-LUC-F | GTCAGGTACCATGAAAGCACCCAACATGGAGAC |
| AtCFL1-LUC-R | GTCAGTCGACTCAAGAAGATGCAGAATGAG |
| CFLAP1-LUC-F | GTCAGGATCCATGGAATCAGAATTCCAGCAACAT |
| CFLAP1-LUC-R | GTCAGTCGACCGCACTAGAGCATCTACATC |
| CFLAP2-TOPO-F | CACCATGCAATCCACTCATATAA |
| CFLAP2-TOPO-R | TTATTGTTCTTCTTTAGGTTTGCATTTGCATCT |
| FBH2-TOPO-F | CACCATGCAACCAACATCCGTCGGTAG |
| FBH2-TOPO-R | TTATTGTTCTTCCTTAGGTATG |
| FBH4-TOPO-F | CACCATGGATTCAAATAATCATCTCTA |
| FBH4-TOPO-R | CTATATTGACTTCTTCTCCTTGTTCATAC |
| AtCFL1 N70 TOPO-R | TCAACGAAAACGACACGAGTTCGAAGTTTC |
| AtCFL1 C119 TOPO-F | CACCATGACAGGAGAGATTTACTACATA |
| AtCFL1 C98 TOPO-F | CACCATGAATGCAGATCCTGACAGT |
| AtCFL1 C77 TOPO-F | CACCATGTATTACGACAGCGAAGAG |
| AtCFL1 C50 TOPO-F | CACCATGGAGGAGGAAGAAGAAGAA |
| AtCFL1 ΔC20 TOPO-R | TCAGTCCTCAACAAGCTTAG |
| AtCFL1C155AC158A-F | GGTTGCTGGTGCCAAAGCTGCCTTCATGTACTTCATGGTTCCTAAG |
| AtCFL1C155AC158A-R | GGCAGCTTTGGCACCAGCAACCACTAGCACATCTTCTTCT |
| AtCFL1C171AC174A-F | CTTGTTGACGCCCCTAAAGCCGCAGCACAGCTTCTTCACT |
| AtCFL1C171AC174A-R | GGCTTTAGGGGCGTCCTCAACAAGCTTAGGAACCATGAAGTA |
| CFLAP1 SRDX-F | GCGCAGATCTATGGAATCAGAATTCCAGCAACATCA |
| CFLAP1 SRDX-R | GCTAACTAGTCGCACTAGAGCATCTACATC |
| CFLAP1 pER8-F | GTCACTCGAGATGGAATCAGAATTCCAGCAACAT |
| CFLAP1 pER8-R | GTCAACTAGTTCACGCACTAGAGCATCTACA |
| CFLAP1-RT-F | CGGACTCCGGTGAATAATCT |
| CFLAP1-RT-R | AAGCGTCCGAGGAGGCAATC |
| CFLAP2-RT-F | CGTTCAGCTCCAGCTACTTG |
| CFLAP2-RT-R | CAGCCGGAGAACTATTCTGA |
| CFLAP1SRDX-RT-F | ACTCATCCTCGAAGCATAGC |
| CFLAP1SRDX-RT-R | CGAAACCCAAACGGAGTTCT |
| FDH-RT-F | GGCTTCTAGGATGACACTTC |
| FDH-RT-R | TTAGAGAGGCACAGGGTAAC |
| BDG-RT-F | ACCATGTCCGTGACAACGTG |
| BDG-RT-R | GTGGATCTGCGCCAAATGAG |
| KCS8-RT-F | CGTTACACGTAGAGGCGTCA |
| KCS8-RT-R | GCAGAGCCACCCATACTGAA |
| DEWAX-RT-F | TTACAGAGGCGTGAGAAGGA |
| DEWAX-RT-R | CATGAATTGACCGGAGCATC |
| TUB2-RT-F | GTTCTCGATGTTGTTCGTAAG |
| TUB2-RT-R | TGTAAGGCTCAACCACAGTAT |
